# A genome-wide association study identifies a horizontally transferred bacterial surface adhesin gene associated with antimicrobial resistant strains

**DOI:** 10.1038/srep37811

**Published:** 2016-11-28

**Authors:** Masato Suzuki, Keigo Shibayama, Koji Yahara

**Affiliations:** 1 Department of Bacteriology II, National Institute of Infectious Diseases, Musashimurayama, Tokyo, 208-0011, Japan

## Abstract

Carbapenems are a class of last-resort antibiotics; thus, the increase in bacterial carbapenem-resistance is a serious public health threat. *Acinetobacter baumannii* is one of the microorganisms that can acquire carbapenem-resistance; it causes severe nosocomial infection, and is notoriously difficult to control in hospitals. Recently, a machine-learning approach was first used to analyze the genome sequences of hundreds of susceptible and resistant *A. baumannii* strains, including those carrying commonly acquired resistant mechanisms, to build a classifier that can predict strain resistance. A complementary approach is to explore novel genetic elements that could be associated with the antimicrobial resistance of strains, independent of known mechanisms. Therefore, we carefully selected *A. baumannii* strains, spanning various genotypes, from public genome databases, and conducted the first genome-wide association study (GWAS) of carbapenem resistance. We employed a recently developed method, capable of identifying any kind of genetic variation and accounting for bacterial population structure, and evaluated its effectiveness. Our study identified a surface adhesin gene that had been horizontally transferred to an ancestral branch of *A. baumannii,* as well as a specific region of that gene that appeared to accumulate multiple individual variations across the different branches of carbapenem-resistant *A. baumannii* strains.

Carbapenems are a class of last-resort antibiotics^1^ that exhibit a broad spectrum of antimicrobial activity, and play a critically important role in medicine. The increase in carbapenem-resistant microorganisms is thus considered one of the most serious public health threats across the world. A species that is becoming increasingly carbapenem-resistant, and which causes severe nosocomial infection worldwide, is *Acinetobacter baumannii*^2^,^3^. Outbreaks of *A. baumannii* frequently occur in healthcare facilities^4^, and it is notoriously difficult to control in hospitals because of its ability to survive for prolonged periods in a wide range of environmental conditions^5^.

*A. baumannii* possesses diverse antimicrobial resistance genes in its chromosome and plasmids^6^. The main mechanism of carbapenem resistance is the production of oxacillinase-type (OXA-type) carbapenemases^7^,^8^. The intrinsic *bla*_OXA-51_ carbapenemase gene, usually encoded on the chromosome, is normally expressed at low levels. However, the insertion of an IS element (IS*Aba1*) in its upstream promoter can drive overexpression and thereby confer resistance to carbapenems^9^. In addition, *A. baumannii* occasionally acquires other OXA-group carbapenemase genes, usually via transposons and plasmids, with *bla*_OXA-23_ being the most commonly acquired, followed by *bla*_OXA-24_ and *bla*_OXA-58_^10^. Until recently, obtaining large numbers of bacterial genomes, with associated metadata on antimicrobial susceptibilities, from public genome databases, was difficult. However, very recently, the genome sequences of 110 carbapenem-susceptible and 122 carbapenem-resistant *A. baumannii* strains were analyzed, and the data were made publicly available in the PATRIC database (Pathosystems Resource Integration Center; www.patricbrc.org)^11^. This study built a machine-learning classifier to predict the resistance of a particular strain, and reported its accuracy to be approximately 95%.

However, this large-scale genomic dataset contained strains encompassing the commonly known mechanisms of carbapenem resistance (for example, the insertion of IS*Aba1* upstream of the *bla*_OXA-51_ carbapenemase gene, and acquisition of the *bla*_OXA-23_ gene). It is not surprising that prediction of carbapenem resistance is possible using commonly known features when these are present in the strains considered. A complementary approach is to explore novel genetic elements, which could be associated with antimicrobial resistance in strains not possessing the commonly known resistance features. Methods for applying genome-wide association studies (GWAS) to bacteria have recently been developed^12^,^13^. In this study, we utilized these methods to identify novel genetic elements associated with carbapenem resistance, and evaluated effectiveness of this approach. We applied the method to genome sequences from carefully selected carbapenem-resistant strains that did not possess the commonly reported resistance features as well as an equivalent number of carbapenem-susceptible strains, spanning various genotypes, from the PATRIC database. We took a kmer-based approach^12^,^13^, in which the genome sequence of each isolate was fragmented into unique, overlapping, 31-bp DNA motifs or kmers, that could be used to identify any kind of genetic variation such as single nucleotide polymorphisms (SNPs), indels, and the presence or absence of a whole gene or gene region. We then explored the DNA motifs that were significantly associated with carbapenem resistance. We accounted for the inter-dependence of the strains and population structure, following a recently developed method^13^ that directly incorporates the relatedness of all of strains and employs statistical tests to examine potential lineage effect as well as a locus effect for a given kmer. Validation and annotation were performed using multiple complete genomes from other strains.

Our study identified a surface adhesin gene that had been horizontally transferred to an ancestral branch of *A. baumannii,* as well as a specific region of that gene that appeared to accumulate multiple individual variations across the different branches of carbapenem-resistant *A. baumannii* strains.

## Results

### DNA motifs associated with carbapenem resistance that are not explained by common mechanisms

**Figure 1:**
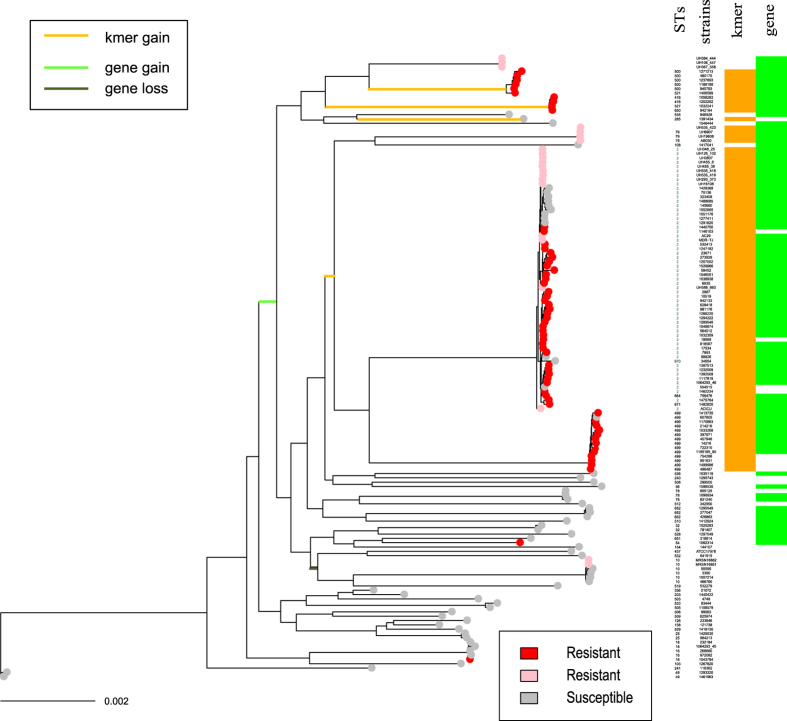
Clonal phylogeny and distribution of the carbapenem-associated genetic elements. The columns “STs” and “strains” between the tree and heatmap indicate sequence type and the name of each strain. STs that were not determined by perfect matching of the alleles are kept blank. Orange and green in the 1^st^ and 2^nd^ columns of the heatmap indicate the presence of a kmer or gene. Red and pink indicate carbapenem-resistant strains used for GWAS discovery and validation, respectively. The branches showing >0.5 probability of kmer gain, gene gain, and gene loss are indicated by orange, green, and black bold lines, respectively. There are several carbapenem-resistant strains that possess the kmer but not the surface adhesin locus. These strains had a shorter BLAST alignment length than was required to define the presence of the gene: 36–48% of the locus length was found in the strains 1146103, 18689, 1462234, 754286, 951631, 1499986, and 496487.

We performed a GWAS using the genome sequences of 61 carbapenem-resistant and 61 carbapenem-susceptible *Acinetobacter baumannii* strains that had neither the *bla*_OXA-23_ gene nor the IS*Aba1* insertion sequence upstream to the *bla*_OXA-51_ gene. Additionally, the selected genome sequences did not contain any other common carbapenemase genes (NDM, IMP, GES, VIM, or KPC). We performed multilocus sequence typing (MLST), developed at the Pasteur Institute^14^, to assign these strains to a total of 46 sequence types (STs); approximately 50% of strains were assigned to ST2, as shown in green in the clonal phylogeny plot in Fig. 1, which has been identified as an international, epidemic, clone^15^,^16^.

During the screening process, we firstly extracted 2,125,002 kmers, fragmented from the genome sequences that were present in over 80% of the resistant strains (red circles in Fig. 1). Within these, we identified 488 kmers that were more than 70% more frequent in the resistant strains than in the susceptible strains (grey circles in Fig. 1); this number was approximately 10% higher than that reported in the previous bacterial GWAS study^12^. Conversely, there were no kmers that occurred over 70% more frequently in the susceptible strains than in the resistant strains.

The statistical significance of the association of carbapenem resistance with each of the candidate kmer was tested as follows. Extraction of 52,363 bi-allelic SNPs in the genomes, and the calculation of an *n* × *n* relatedness matrix that summarizes all genetic covariance among the strains, was performed to control for background population structure. Using the linear mixed regression model in the R package *bugwas*^13^, which uses the relatedness matrix to model the background random effect, we found that the association was highly significant for 469 out of 488 kmers after false discovery rate (FDR) correction (P_FDR_ < 0.05).

Before testing the statistical significance of the kmers using linear-mixed regression, principal component analysis was conducted using the *bugwas* package to test for potential lineage effects. However, no principal component was found to be significantly associated with carbapenem resistance. This suggests that the significant association between the kmer and carbapenem resistance was not confined to a specific lineage but was rather observed across multiple lineages. Further evidence for this interpretation is provided below.

### Discovery of a putative adhesin gene

Locations of the 469 statistically significant kmers were searched against the complete genome sequences of a carbapenem-susceptible strain (ATCC 17978), and four carbapenem-resistant strains (AB030, AC29, ACICU, and MDR-TJ). Of these, 212 kmers were mapped to genes of at least one of the genomes of resistant strains, but not to those of the susceptible strains (Table S1). All of the kmers corresponded to 15 genes (Table 1) that have nonsynonymous variations compared to the susceptible genome. Most of these genes are related to metabolism or nutrient transport, and broad housekeeping functions. For example, a previous study that analyzed single-gene deletion mutants of *Acinetobacter baylyi* ADP1 demonstrated that *rph*, which encodes the ribonuclease PH, and ACICU_00262, which encodes homoserine dehydrogenase, were essential for survival^17^.

In addition, the gene ACICU_02910, which encodes a putative surface adhesin, may facilitate adhesion to host cells and is expected to have a larger contribution to fitness of the resistant strains compared to the housekeeping genes. The putative surface adhesin, consisting of 3169 amino acids, is encoded at nucleotide positions 3,076,290–3,085,799 on the minus strand of the ACICU genome. Overall, 79 out of the 212 kmers were mapped to the locus of at least one of the four resistant complete genomes (AB030, AC29, ACICU, and MDR-TJ).

Prediction of the structure and function of the ACICU_02910 gene product, using the PHYRE protein fold recognition server^18^, revealed that the N-terminal amino acids at positions 138–523 (encompassing the kmers located at nucleotide positions 1374–1406) were modeled with 99.9% confidence to the highest scoring protein template: a Ca^2+^-stabilized adhesin of unknown function (PDB ID 4P99). Three out of the 79 kmers were mapped to all four resistant complete genomes (AB030, AC29, ACICU, and MDR-TJ). The kmers were mutually overlapping and located within the adhesin-associated region at nucleotide positions 1374–1406 within the locus. Moreover, the C-terminal amino acids in positions 2952–3149, which include the Ca^2+^-binding domain (pfam00353 in NCBI CDD) and type I secretion C-terminal target domain (TIGR03661 in NCBI CDD), were modeled with 99.8% confidence to the highest scoring protein template: the Serralysin-like metalloprotease, C-terminal domain (SCOP ID d1kapp1). BLAST searching using the peptidase database MEROPS^19^ predicted that the ACICU_02910 protein did not possess any typical protease catalytic domains, including those of metallo-types; ACICU_02910 was instead classified into the unknown catalytic family U69, containing self-processing peptidases such as adhesin AIDA-I in *E. coli*^20^, the extracellular portion of which is autocatalytically released. AIDA-I mediates self-association and biofilm formation^21^, as well as invasion of epithelial cells. An NCBI CDD search also revealed several regions encoding bacterial Ig-like domains, one of which is located at the amino acid positions 1184–1245.

**Figure 2:**
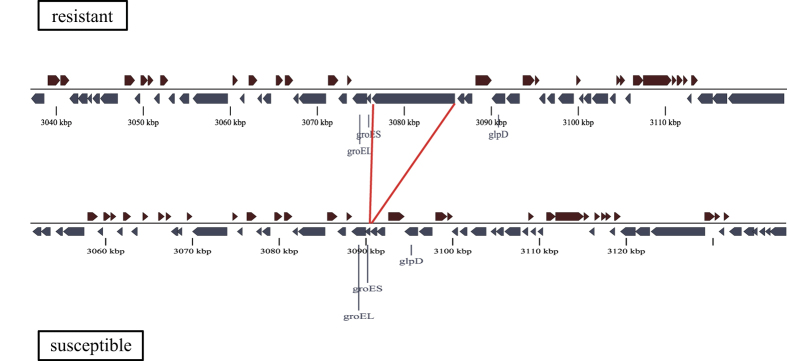
Genomic context of the adhesin gene. The upper panel shows the resistant strain ACICU, while the lower panel shows the susceptible strain ATCC 17978. The adhesin gene (ACICU_02910) is absent in the susceptible strain.

**Figure 3:**
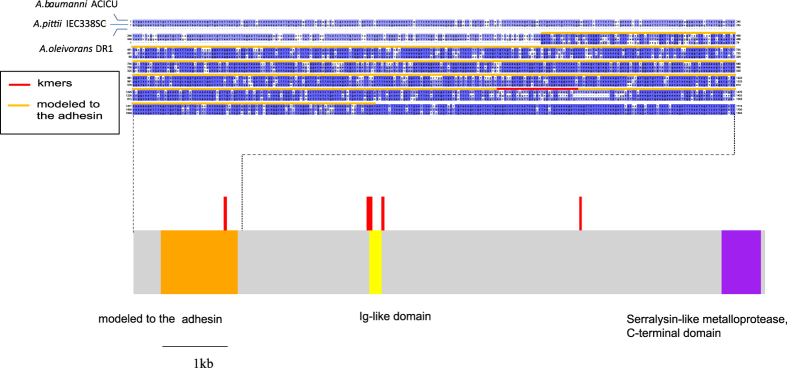
Locations of regions encoding the domains and carbapenem-associated kmers in the adhesin gene, and between-species alignment of nucleotide sequences adjacent to the adhesin-associated region. The red vertical lines indicate the statistically significant kmers that were mapped to the genome of at least one of the four resistant strains (AB030, AC29, ACICU, and MDR-TJ), including the three overlapping kmers that mapped to the adhesin-associated region of all four genomes (orange). The red and orange horizontal lines in the alignment indicate the positions of the three overlapping kmers, and the predicted Ca^2+^-stabilized adhesin, respectively.

Figure 2 shows the genomic context of the adhesin gene and its surrounding genes in the resistant ACICU and the susceptible ATCC 17978 strains. The gene is approximately 9.5 kb in size. The specific intragenic locations of the kmers mapping to the adhesin gene in at least one of the four resistant strains (AB030, AC29, ACICU, and MDR-TJ) are shown in the lower part of Fig. 3. Three of the kmers that mapped to all four complete genomes of resistant strains were located in the adhesin-associated region (orange in Fig. 3). Presence or absence data of the gene and the three overlapping kmers are shown in the heatmap on the right of Fig. 1. Presence of the gene was defined as a BLAST match of ≥70% identity, over ≥50% of the locus length^22^.

### Horizontal transfer of the adhesin gene

Figure 2 shows that the gene is obviously larger than the surrounding genes. It was predicted to be a horizontally transferred “alien” gene by the Alien_Hunter application^23^, which identifies atypical nucleotide compositions based on variable order motif distributions. The topology of the phylogenic tree based on the conserved portions of the gene (nucleotide positions from 544 to the end within the locus) was clearly different from the clonal genealogy (Fig. S1, indicated by the notable incongruence of the lines). The size of the homologously recombined fragments surrounding the gene, inferred from the clonal phylogeny of *A. baumannii* (Fig. 1), was at most 2.7 kb, which cannot explain the import of the “alien” gene. We also used the TreeBreaker^24^ model to infer the probability of having a changepoint of carriage of the gene on each branch; branches with a posterior probability >0.5 are indicated in green or as a black bold line in Fig. 1. The results showed that the gene was initially acquired on the ancestral branch of *A. baumannii,* and was then maintained in most of the resistant strains, but was lost in some lineages of susceptible strains.

### Distribution of the adhesin gene in the *Acinetobacter calcoaceticus*-*baumannii* (*Acb*) complex

A BLAST search of the nucleotide sequence against bacterial entries in GenBank revealed that the 2^nd^ and 3^rd^ hits (following the top hit, *A. baumannii*) were the *Acinetobacter pittii* strain IEC338SC and *Acinetobacter oleivorans* strain DR1, with 89% and 88% sequence identity over 100% and 95% of the alignment length of the locus, respectively. A sequence alignment based on the BLAST search is shown in the upper part of Fig. 3. Although the 411 bases from the 5′ end of the 3^rd^ hit, *Acinetobacter oleivorans* DR1, are not aligned, the other bases form an alignment across the locus, including the regions mapped by the most strongly associated kmers (nucleotide positions 1374–1406, indicated by a red line), and those modeled using the Ca^2+^-stabilized adhesin protein template (amino acid positions 138–523, indicated by an orange line). Overall alignment identity between each of the three sequences was 88.7% (between 1^st^ and 2^nd^), 88.1% (between 1^st^ and 3^rd^), and 93.2% (between 2^nd^ and 3^rd^), respectively.

**Figure 4:**
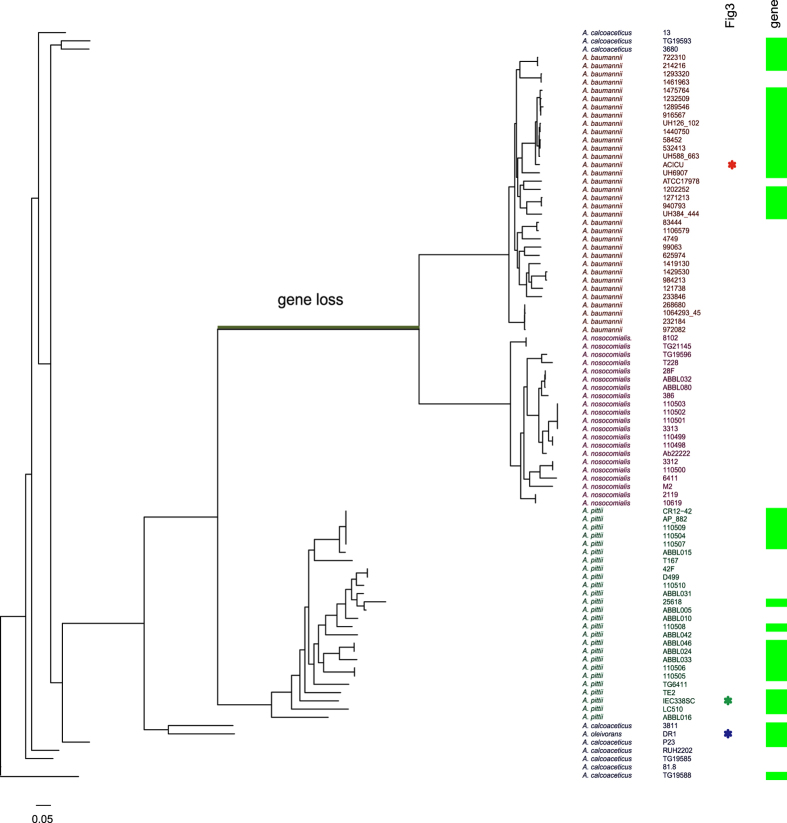
Phylogeny of *Acinetobacter calcoaceticus*-*baumannii* (*Acb*) complex and distribution of the adhesin gene. The green squares indicate the presence of the adhesin gene. The three strains shown in Fig. 3 are indicated by asterisks. The branches showing a >0.5 probability of gene loss are indicated with black bold lines.

The 2^nd^ hit, *A. pittii*, is a nosocomial pathogen similar to *A. baumannii*^25^, and forms the monophyletic *A. calcoaceticus*-*baumannii* (*Acb*) complex along with *A. baumannii* and two other closely related species: *A. nosocomialis* and *A. calcoaceticus*^26^. The 3^rd^ hit, *Acinetobacter oleivorans* DR1, is phylogenetically included in the same clade as *A. calcoaceticus*^26^, and thus forms part of the *Acb* complex. A phylogeny of the *Acb* complex, along with presence or absence of the adhesin gene, is shown in Fig. 4. The phylogeny includes the 2^nd^ and 3^rd^ hit strains, other strains in the three non-*baumannii* clades registered in the PATRIC database, and 34 additional *A. baumannii* strains, which were selected from the lineages (Fig. 1) as representatives of the carbapenem-resistant and -susceptible strains. We found several strains of *A. pittii* and *A. calcoaceticus* that carry the adhesin gene, although no *A. nosocomialis* strains were found to carry the gene. The TreeBreaker^24^ model showed that loss of the gene occurred with posterior probability >0.5 on a branch (bold black in Fig. 4) ancestral to *A. baumannii* and *A. nosocomialis*.

### Association of the DNA motifs on the adhesin gene is observed in multiple lineages

Broadly, the phylogenetic tree in Fig. 1 separates the isolates into those in clonal groups that are mostly resistant and contain the kmers and genes, and those in long branches that are mostly sensitive and do not have the kmer and genes. The extent of association was found to be weaker for the entire gene than for the kmers, which can be explained by the ancestral gain, as inferred by TreeBreaker analysis (green branch in Fig. 1) and subsequent clonal inheritance. In fact, the test of the locus effect for the entire gene rather than for the DNA motifs was not significant after correcting for population structure.

In contrast, regarding the significant kmers on the adhesin gene, the TreeBreaker model inferred that the kmer gains occurred multiple times on different branches showing a probability of kmer gain >0.5 (orange branches in Fig. 1). The pattern of recurrent evolutionary signals across different branches suggests that the presence of the kmer could be generally advantageous for carbapenem-resistant populations.

We performed validation by investigating whether the three kmers on the adhesin gene were frequently present in the genome sequences of other carbapenem-resistant strains. In addition to the four complete genomes described above (AB030, AC29, ACICU, and MDR-TJ), we used 18 genomes from the PATRIC database that were annotated as carbapenem-resistant, all of which are colored pink in the clonal phylogeny (Fig. 1). Approximately 80% of the genomes of carbapenem-resistant strains have the kmer, as shown in the 1^st^ column of the heatmap in Fig. 1. Among the 23 genomes (colored pink in Fig. 1), only the AB030 strain has acquired an *bla*_OXA-23_-like carbapenemase gene.

## Discussion

This is the first bacterial GWAS study to focus on carbapenem-resistant strains that lack the commonly acquired resistance mechanisms, in the hope of revealing novel genetic elements associated with carbapenem resistance, such as SNPs, indels, or the presence or absence of genes or gene regions as determined by the kmer-based approach. We found multiple candidates (Table 1), and demonstrated that the strongest and most interesting GWAS hit kmer corresponded to the presence of a specific region encoding the adhesin, similar to the previous bacterial GWAS study that revealed a specific gene corresponding to significant kmers^12^. The gene was judged to be “alien” because it showed an atypical nucleotide composition compared with other genomic regions, indicating that it was probably horizontally transferred from a distant relative of the *Acb* complex.

The adhesin gene was found to be present across all clades of the *Acb* complex, except for the *A. nosocomialis* lineage. The TreeBreaker model inferred that the gene was lost on a branch ancestral to *A. baumannii* and *A. nosocomialis*, but was acquired at an ancestral branch in *A. baumannii*. After correcting for the population structure, a statistically significant association was found not for the entire gene but rather for the kmers mapped on the adhesin gene. Application of the TreeBreaker model to the pattern of presence or absence of the kmers revealed that after the acquisition of the gene in *A. baumannii*, the carbapenem-associated genetic elements on the adhesin gene were gained multiple times on different branches, indicating a dynamic evolutionary process involving selective forces, and providing statistical evidence of the favorability of these genetic elements.

However, there are caveats concerning both the identification of the adhesin gene and the evaluation of its significance. Firstly, it does not possess any of the protein domains typically associated with β-lactam resistance. Secondly, although the carbapenem resistance phenotype is binary and strains are unambiguously either susceptible or resistant, several strains that possess both the adhesin and the key kmer remain susceptible to carbapenem (Fig. 1). It is thus unlikely that the putative adhesion function directly causes antimicrobial resistance, and precisely how the resistant strains acquired their carbapenem resistance without the commonly known mechanisms remains largely unknown. Other mechanisms for enabling overexpression of the intrinsic *bla*_OXA-51_ carbapenemase gene in resistant strains, but not in susceptible strains, may exist. Such mechanisms, however, would not be universally shared among resistant strains. Another study, based on gene expression data, will be required for further investigation.

The peptidase database MEROPS classified the adhesin gene as belonging to the U69 family. Another U69 family member, the *E. coli* AIDA-I autotransporter protein, mediates biofilm formation, which has been implicated in antimicrobial resistance and bacterial survival in the presence of antibiotics^27^,^28^. Although the relationship between biofilm formation and antimicrobial resistance in *A. baumannii* was, until recently, poorly understood, a transcriptomic study revealed a negative association between carbapenem resistance and biofilm production, wherein gene groups involved in biofilm formation were downregulated in the presence of carbapenem^29^. Another study, using 116 strains, detected an inverse relationship between meropenem resistance and biofilm production^30^. A more recent study revealed that biofilms formed by highly resistant strains were always weaker than others; however, analysis of the minimum biofilm eradication concentration showed that, once formed, these biofilms showed a similar level of enhanced antimicrobial resistance^31^. These findings suggest an unknown genetic mechanism that enables resistant strains to achieve high levels of biofilm-specific resistance, despite producing weak biofilms. The adhesin gene found in this study could play such a compensatory role in resistant strains. Further laboratory studies are warranted to obtain validation of its function, which will require the collection of strains carrying the gene and establishment of site-directed mutants at the target adhesin-associated region identified in this study.

Generally, the strengths of the bacterial GWAS methods reported to date lie in the identification of any kind of genetic variation such as SNPs, indels, or the presence or absence of genes or gene regions by using a kmer approach. They do not require a single reference genome but take a reference-free approach, similar to a recently reported machine-learning study. Additionally, bacterial GWAS approaches enable us to control for the strong inter-dependence and population structure between strains, illustrated by their phylogeny. In contrast, the machine-learning approach does not consider the inter-dependence of individuals, which is not necessarily required if the purpose is simply to build a machine-learning classifier. In order to conduct a GWAS rather than a machine-learning approach, controlling for the inter-dependence of strains, or for population structure, is necessary to avoid inflation of the type I error, as described in previous reports of bacterial GWAS approaches^12^,^32^,^33^,^34^, one of which also took a machine-learning approach and succeeded in building a classifier of virulence^32^. We utilized the method implemented in the *bugwas* package, which, unlike a pioneering bacterial GWAS method based on clonal phylogeny^12^, does not involve splitting the data into each clonal complex for separate analysis, thus reducing the sample size for GWAS discovery.

Nonetheless, bacterial GWAS approaches should be undertaken with a note of caution. It can be difficult to identify a specific gene that corresponds to significant kmers if, for example, the kmers do not map to any of the reference genomes. Even when a specific gene can be identified, the degree of association between the kmers and the gene can be variable, and may only be statistically significant for a region of a gene, as was seen in the present study. Moreover, an association other than the desired direct causal effect could occur. For example, it is possible that strains from patients with severe infectious disease, which may bear specific virulence determinants such as an adhesin, are isolated more frequently than other strains. If patients have previously been treated with a range of antibiotics, it is likely that infections caused by antibiotic-susceptible strains would be resolved, and thus these strains would not have been sampled. The recovered strains are therefore more likely to be resistant, resulting in the association between resistance and the virulence determinant.

In the future, the potential of this GWAS approach can be maximized by using more genomic sequences from various clades, with associated metadata encompassing antimicrobial susceptibilities and the clinical or environmental conditions in which the strains were sampled. This allows case and control strains to be matched both phylogenetically and according to their clinical or environmental metadata. Overall, careful preparation of datasets is advisable when using this GWAS approach.

## Materials and Methods

### Selection of *A. baumannii* strains for discovery and validation in GWAS

Sixty-one carbapenem-resistant and sixty carbapenem-susceptible strains were selected from a recently published dataset of *Acinetobacter baumannii* genome sequences^11^. These strains all had the intrinsic *bla*_OXA-51_ gene, but lacked the *bla*_OXA-23_ gene, the IS*Aba1* insertion sequence upstream of the *bla*_OXA-51_ gene, or any other common carbapenemase genes (NDM, IMP, GES, VIM, or KPC). The complete genome of the carbapenem-susceptible ATCC 17978 strain^35^, which satisfied the conditions above, was also used.

To detect carbapenemase genes, we conducted a BLAST search of every locus in the ResFinder^36^ and ARG-ANNOT^37^ databases against each genome, and used a BLAST match of ≥70% identity, over ≥50% of the locus length, as the criteria for positive detection of the gene. To detect the IS*Aba1* insertion sequence upstream of the *bla*_OXA-51_ gene, we extracted a 2012-nucleotide sequence from a previously reported plasmid (GenBank accession no. GQ352402)^38^, and then conducted a BLAST search against each genome, using a BLAST match of ≥90% identity, over ≥90% of the length, as a positive indication of its presence.

Additionally, we used other *A. baumannii* genomes that were annotated as carbapenem resistant in the PATRIC database, and that satisfied the conditions above, for validation. In addition, from the 20 complete genomes of *A. baumannii* listed in a recent report^39^, we used those of carbapenem-resistant strains ACICU^40^, MDR-TJ^41^, AB030^42^, and AC29^10^.

MLST typing of the strains was conducted by perfectly matching the allelic sequences defined in the Pasteur scheme^14^. The STs and the strain names are shown in Fig. 1.

Within the original dataset hosted in the PATRIC database^11^ (ftp://ftp.patricbrc.org/patric2/current_release/AMR_genome_sets), we found that some strains were phylogenetically quite distant from other strains (dashed circle in Fig. S2). Although they were defined as *Acinetobacter baumannii*, we considered that they may in fact belong to different *Acinetobacter* species. In fact, some of the strains did not carry an *bla*_OXA-51_ gene (2^nd^ column of the heatmap in Fig. S2), this being the intrinsic carbapenemase gene in *Acinetobacter baumannii*. We thus excluded these strains from our analyses.

### Selection of non-*A. baumannii* strains in the *Acb* complex

We used all of the genomic sequences of the *A. pittii, A. nosocomialis,* and *A. calcoaceticus* strains that were registered in the PATRIC database, with the exception of some strains that were annotated to one species yet clustered with strains of a different species in a core-genome phylogeny.

### Construction of the phylogeny

We constructed a concatenated core-genome alignment using the Roary pipeline^43^, and then a maximum-likelihood tree either for the GWAS dataset (Fig. 1) or for the *Acb* complex (Fig. 4) using PhyML^44^. Using this as the starting tree, we constructed a clonal phylogeny, with corrected branch lengths to account for homologous recombination, using ClonalFrameML^45^. We used the extended model, which allows for different recombination parameters to be inferred on different branches of the clonal phylogeny. In order to infer homologous recombination events in both the core and non-core regions, we used a whole genome alignment as an input, with the ACICU genome as the reference, and constructed gene-by-gene alignments using BLAST and MAFFT^46^, before combining them at relevant positions on the reference genome. We included sites that were missing in less than 40% of strains in the alignment.

Likewise, we constructed a core-genome alignment followed by a maximum-likelihood tree for the GWAS dataset using the FastTree application^47^, in order to examine the original dataset (Fig. S2). This application was selected instead of PhyML to reduce the computational time and to mainly understand the topology of the phylogenetic tree.

### Kmer-based GWAS to detect either lineage or locus effects after accounting for population structure

For each strain, we listed all of the unique 31-bp DNA motifs, or kmers, in its genome, using the dsk software^48^. We then calculated the difference in the frequency of appearance of each kmer in the carbapenem-resistant and -susceptible populations, and extracted kmers with a more than 70% frequency difference. Meanwhile, we extracted bi-allelic SNPs from the concatenated core-genome alignment created using Roary. The bi-allelic SNPs were then used to conduct principal component analysis to test for potential lineage effects, and to calculate an *n* × *n* relatedness matrix to be used in a linear mixed regression with the *bugwas* package. It incorporates the *gemma*^49^ program to use the linear mixed regression model, test for the locus effects, and estimate parameters to test for the potential lineage effects in the *bugwas* package. We downloaded and compiled the source code for *gemma*^49^ (version 0.95a) from GitHub (https://github.com/xiangzhou/GEMMA)

### Characterization of the gene and DNA motifs found by GWAS

The amino acid sequence was annotated using the PHYRE protein fold recognition server^18^ and the peptidase database MEROPS^19^. The nucleotide sequence was searched against bacterial entries in GenBank, using Blast+ with the default settings (match reward = 2). The genomic context was visualized and examined using GView^50^. Atypical nucleotide compositions, which could indicate horizontal gene transfer, were investigated further using Alien_Hunter^23^. The probability of having a changepoint of carriage of the gene and kmers was inferred on each branch in the phylogeny using TreeBreaker^24^, which is based on the principle of an evolving property (in our case, the presence or absence of the gene or kmer) distribution on the branches of a phylogeny. All branches with a probability >0.5 are indicated with bold lines in the phylogenies.

## Additional Information

**How to cite this article**: Suzuki, M. *et al*. A genome-wide association study identifies a horizontally transferred bacterial surface adhesin gene associated with antimicrobial resistant strains. *Sci. Rep.* **6**, 37811; doi: 10.1038/srep37811 (2016).

**Publisher's note:** Springer Nature remains neutral with regard to jurisdictional claims in published maps and institutional affiliations.

## Supplementary Material

Supplementary Information

## Figures and Tables

**Table 1:** Genes carrying the carbapenem-associated genetic variations.

Locus tag*	Description
ACICU_02910	putative surface adhesin protein
ACICU_00262	homoserine dehydrogenase
ACICU_00264	site-specific recombinase XerD
ACICU_00272	predicted phosphohydrolase
ACICU_00865	hypothetical protein
ACICU_01307	ATPase with chaperone activity, ATP-binding subunit
ACICU_01366	purine-cytosine permease
ACICU_01449	dehydrogenase with different specificities
ACICU_02301	ethanolamine ammonia-lyase, small subunit
ACICU_02437	gamma-aminobutyrate permease
ACICU_02439	adenosylmethionine-8-amino-7-oxononanoate aminotransferase
BL01_01510	*rph*, ribonuclease PH
ABTJ_03740	alpha-hydroxyacid dehydrogenase, FMN-dependent l-lactate dehydrogenase
ABTJ_02588	hypothetical protein
ABTJ_02897	hypothetical protein

All of the variations are nonsynonymous compared to the susceptible genome ATCC17978. Locus tags are shown for the genes of the carbapenem-resistant strains (ACICU, AC29, and MDR-TJ) onto which the kmers were mapped.
