# Supplementary material for: A genome-wide association study identifies a horizontally transferred bacterial surface adhesin gene associated with antimicrobial resistant strains
Source: Sci Rep. 2016 Nov 28;6:37811. doi: 10.1038/srep37811 (PMC5124939; doi:10.1038/srep37811)
Supplement: Supplementary Information [file srep37811-s1.pdf]

Supplemental Information for:

A genome-wide association study identifies a horizontally transferred bacterial surface adhesin gene associated with antimicrobial resistant strains

Masato Suzuki[1], Keigo Shibayama[1], and Koji Yahara[1*]
[1] Department of Bacteriology II, National Institute of Infectious Diseases,

Musashimurayama, Tokyo, 208-0011, Japan

[*]Corresponding author: Koji Yahara (k-yahara@nih.go.jp)

Supplementary text

1. Reproducibility

We prepared a shell script that can reproduce the GWAS results. The script file and its input and dependent files are downloadable at https://1drv.ms/u/s!AgviESvFRNpSgxm26yJU1UHtCz4R

It uses following external software: bugwas, gemma, R, dsk, plink, blast, perl and BioPerl. Software versions we tested are as follows.

software	version
bugwas (R package)	1.0
gemma	0.95alpha compiled from the latest source code in GitHub, or 0.93beta incorporated in bugwas package
R	3.3.1
ape (R package)	3.5
adegenet (R package)	2.0.1
dsk	2.1.0
plink	1.0.7
blast	2.2.25
perl	5.14.2
BioPerl	1.6.923

All binary files except for bugwas must be installed in your PATH. If you use the SGE/UGE grid computing system where qsub command is available, the script automatically detects it and submits jobs for which at most 64GB RAM is allocated.

Regarding bugwas, we slightly modified BUGWAS_functions.R and genVarPlots.R to handle strain IDs starting numbers rather than alphabets, and add additional information (e.g, FDR-adjusted p-values) to its output. Please "unzip bugwas.zip" in the downloaded files, and install it by "R CMD INSTALL bugwas".

The script takes the following command-line arguments:

-g	file of genome-wide alignment (fasta format)
-f	directory where genome sequences of individuals are stored as ID.fna files
-p	file where 1st column is ID and 2nd column is pheno (0 or 1)
-c	cutoff of frequency change (kmers showing frequency change less than

	this value are filtered out)
-m	(optional)<br>cutoff of the number of case individuals (kmers present in less than the number of case individuals are filtered out in advance)
-t	tree file (newick format)
-a	directory where *.fna, *.ffn, and *.gff files (output files of prokka) or *.chrseq, *.geneseq, and *.gene files (available at MBGD) are stored for annotation
-o	prefix of final output files

An example to execute the script by using the input files downloaded by the link above is

```
/bin/bash exec_lin_loc_pipeline.sh ¥
-g core_gene_alignment.aln.fas ¥
-f fna/ ¥
-p kmer_dir.pheno.ID.txt ¥
-c 0.7 -m 49 ¥
-t tree.nwk ¥
-a annotation/ ¥
-o out_GWAS
```

Source codes of the script are shown in the next page.

```
#! /bin/bash
#$ -cwd
#$ -S /bin/bash

usage () {
  echo "/bin/bash $0 "
  echo " -g core_gene_alignment.aln.fas "
  echo " -f fna/ "
  echo " -p kmer_dir.pheno.ID.txt "
  echo " -c 0.7 [-m 49] "
  echo " -t tree.nwk "
  echo " -a annotation/ (where *.chrseq, *.geneseq, *.gene files or *.fna, *.ffn, *.gff files are)"
  echo " -o out_prefix "
  exit 1
}

echo_fail(){
  echo "Failed: `date '+%Y%m%d_%H%M%S'` $*"
  exit 1
}

MIN_IN_CASE_POP=0
while getopts g:f:p:c:m:t:a:o: OPTION
do
  case $OPTION in
    g)  if [ ! -z "${OPTARG}" ];then CORE_GENOME_FAS=${OPTARG} ;else usage ;fi
        ;;
    f)  if [ ! -z "${OPTARG}" ];then FNA_DIR=${OPTARG} ;else usage ;fi
        ;;
    p)  if [ ! -z "${OPTARG}" ];then PHENO_FILE=${OPTARG} ;else usage ;fi
        ;;
    c)  if [ ! -z "${OPTARG}" ];then CUTOFF_FREQ=${OPTARG} ;else usage ;fi
        ;;
    m)  if [ ! -z "${OPTARG}" ];then MIN_IN_CASE_POP=${OPTARG} ;else usage ;fi
        ;;
    t)  if [ ! -z "${OPTARG}" ];then TREE_FILE=${OPTARG} ;else usage ;fi
        ;;
    a)  if [ ! -z "${OPTARG}" ];then ANNOTATION_DIR=${OPTARG} ;else usage ;fi
        ;;
    o)  if [ ! -z "${OPTARG}" ];then OUT_PREFIX=${OPTARG} ;else usage ;fi
        ;;
    ¥?) usage ;;
  esac
done

if [ $# -lt 14 ] ; then
  usage
fi


FNA_DIR=`echo ${FNA_DIR} | perl -pe 's/¥/$//g'`

if [ ! -s "${CORE_GENOME_FAS}" ]; then
  echo_fail "Error: ${CORE_GENOME_FAS} was not found"
fi

if [ ! -d "${FNA_DIR}" ]; then
  echo_fail "Error: ${FNA_DIR} was not found"
fi

if [ ! -s "${PHENO_FILE}" ]; then
  echo_fail "Error: ${PHENO_FILE} was not found"
fi

if [ ! -s "${TREE_FILE}" ]; then
  echo_fail "Error: ${TREE_FILE} was not found"
fi

if [ ! -d "${ANNOTATION_DIR}" ]; then
  echo_fail "Error: ${ANNOTATION_DIR} was not found"
fi


#############################################################
# check environmental settings
#############################################################

QSUB_ENV=0
CHECK=`which qsub > /dev/null 2>&1`
if [ $? -eq 0 ]; then
  QSUB_ENV=1
fi

arr_bin=(
  dsk
  blastall
  formatdb
```

```
  plink
  gemma
  R
)

for aa in ${arr_bin[@]}
do
  CHECK=`which ${aa}`
  if [ "${CHECK}" == "" ]; then
    echo_fail "Error: ${aa} is not available"
  fi
done


############################################################
# functions
############################################################

get_stamp() {
  DATE_N=`date +%N`
  TIME_DIGIT=`echo "$(date +%H%S)$(printf '%02d' $(expr $DATE_N / 10000000))"` # hour(2)sec(2)milisec(2)
  #if ls "s$1_${TIME_DIGIT}"* &> /dev/null; then
  while ls "s$1_${TIME_DIGIT}"* &> /dev/null
  do
    sleep 1
    DATE_N=`date +%N`
    TIME_DIGIT=`echo "$(date +%H%S)$(printf '%02d' $(expr $DATE_N / 10000000))"` # hour(2)sec(2)milisec(2)
  done
  #fi
  STAMP="s$1_${TIME_DIGIT}"
}

wait_until_finish() {
  if [ "${QSUB_ENV}" -eq 1 ]; then
    while :
    do
      QSTAT_CMD=qstat
      END_CHECK=`${QSTAT_CMD} | grep -e $1 -e 'not ready yet' | wc -l`
      if [ "${END_CHECK}" -eq 0 ]; then
        break
      fi
      sleep 3
    done
  fi
}

disp_punctuate() {
  echo "*************** STEP$1 ***************************** "
  date +%Y%m%d_%T
}

abs_dirname() {
  local cwd="$(pwd)"
  local path="$1"

  while [ -n "$path" ]; do
    cd "${path%/*}"
    local name="${path##*/}"
    path="$(readlink "$name" || true)"
  done

  pwd -P
  cd "$cwd"
}

SCRIPT_DIR="$(abs_dirname "$0")"



############################################################
# variables
############################################################

#
# output
#
DIR_KMER=kmer_dir

OUT_STEP3_FAS=out_assoc_candidate_words.m${MIN_IN_CASE_POP}.c${CUTOFF_FREQ}.fas
OUT_STEP3_VAR=out_assoc_candidate_words.m${MIN_IN_CASE_POP}.c${CUTOFF_FREQ}.fas.var.matrix.txt

#
# scripts
#
PL_COUNT_KMER_EACH=${SCRIPT_DIR}/count_kmers_each_pop.pl
```

```
PL_COUNT_KMER_ACROSS=${SCRIPT_DIR}/count_kmers_across_pops_creating_var_matrix.pl

R_SNP_CALL=${SCRIPT_DIR}/chromo_extract_2allele_noNaSNP_matrix_SI.R
PL_CONVERT_SNP2PLINK=${SCRIPT_DIR}/convert_adegenetMatrix2PlinkBugwas.pl

PL_ANNOTATE=${SCRIPT_DIR}/annotate_words_bugwas.pl



################################################################################
# create kmers of each individual
################################################################################
STEP=1
disp_punctuate ${STEP}

if [ -d "${DIR_KMER}" ]; then
  echo "${DIR_KMER} already exists.  STEP${STEP} was skipped."
else
  mkdir ${DIR_KMER}

  JOB_NAME=dsk
  for aa in `ls ${FNA_DIR}/*.fna`
  do
    CMD="dsk -file ${aa} -kmer-size 31 -abundance-min 1"
    echo ${CMD}

    if [ "${QSUB_ENV}" -eq 1 ]; then
      qsub -cwd -S /bin/bash -N ${JOB_NAME} -l s_vmem=16G -l mem_req=16G -e /dev/null -o /dev/null <<< "
        ${CMD}
      "
    else
      eval ${CMD}
      if [ $? -ne 0 ]; then
        echo_fail "Error: ${CMD}"
      fi
    fi
  done
  wait_until_finish ${JOB_NAME}


  JOB_NAME=dsk2ascii
  for aa in `ls *.h5`
  do
    bb=`echo ${aa} | perl -pe 's/¥.h5//g'`
    CMD="dsk2ascii -file ${bb}.h5 -out ${bb}.kmer31"
    echo ${CMD}

    if [ "${QSUB_ENV}" -eq 1 ]; then
      qsub -cwd -S /bin/bash -N ${JOB_NAME} -e /dev/null -o /dev/null <<< "
        ${CMD}
      "
    else
      eval ${CMD}
      if [ $? -ne 0 ]; then
        echo_fail "Error: ${CMD}"
      fi
    fi
  done
  wait_until_finish ${JOB_NAME}


  JOB_NAME=gzip
  for aa in `ls *.h5`
  do
    bb=`echo ${aa} | perl -pe 's/¥.h5//g'`
    CMD="gzip ${bb}.kmer31"
    echo ${CMD}

    if [ "${QSUB_ENV}" -eq 1 ]; then
      qsub -cwd -S /bin/bash -N ${JOB_NAME} -e /dev/null -o /dev/null <<< "
        ${CMD}
      "
    else
      eval ${CMD}
      if [ $? -ne 0 ]; then
        echo_fail "Error: ${CMD}"
      fi
    fi

    /bin/rm ${aa}
  done
  wait_until_finish ${JOB_NAME}


  CMD="/bin/mv *.kmer31.gz ${DIR_KMER}"
  echo ${CMD}
```

```
  eval ${CMD}
  if [ $? -ne 0 ]; then
    echo_fail "Error: ${CMD}"
  fi
fi

WC_DIR_KMER=`ls ${DIR_KMER}/*.gz | wc -l`
if [ "${WC_DIR_KMER}" -eq 0 ]; then
  echo_fail "Error: there is no *.gz file in ${DIR_KMER}"
fi

################################################################################
# count the kmers for each population
################################################################################
STEP=2
disp_punctuate ${STEP}

if [ -s "kmer_count_group0.txt" -a -s "kmer_count_group1.txt" ]; then
  echo "kmer_count_group0.txt and kmer_count_group1.txt files already exists.  STEP${STEP} was skipped."
else

  perl -i -pe 's/¥.f[a-z]+¥t/¥t/g' ${PHENO_FILE}

  JOB_NAME=step2

  CMD="perl ${PL_COUNT_KMER_EACH} -p ${DIR_KMER}/ -l ${PHENO_FILE} "
  echo ${CMD}

  if [ "${QSUB_ENV}" -eq 1 ]; then
    qsub -cwd -S /bin/bash -N ${JOB_NAME} -l s_vmem=16G -l mem_req=16G  -e /dev/null -o /dev/null <<< "
      ${CMD}
    "
  else
    eval ${CMD}
    if [ $? -ne 0 ]; then
      echo_fail "Error: ${CMD}"
    fi
  fi
  wait_until_finish ${JOB_NAME}
fi

if [ ! -s "kmer_count_group0.txt" ]; then
  echo_fail "Error: kmer_count_group0.txt was empty after STEP${STEP}"
fi

if [ ! -s "kmer_count_group1.txt" ]; then
  echo_fail "Error: kmer_count_group1.txt was empty after STEP${STEP}"
fi


################################################################################
# extract kmers that are more than ${CUTOFF_FREQ} more frequent
################################################################################
STEP=3
disp_punctuate ${STEP}

if [ -s "${OUT_STEP3_FAS}" -a -s "${OUT_STEP3_VAR}" ]; then
  echo "${OUT_STEP3_FAS} and ${OUT_STEP3_VAR} already exists.  STEP${STEP} was skipped."
else

  KMER_CONTROL=kmer_count_group0.txt

  KMER_CASE=kmer_count_group1.txt
  if [ "${MIN_IN_CASE_POP}" -gt 0 ]; then
    KMER_CASE=kmer_count_group1.min${MIN_IN_CASE_POP}.txt

    awk -F '¥t' "{if(¥$2>=¥"${MIN_IN_CASE_POP}¥"){print ¥$0}}"  kmer_count_group1.txt > ${KMER_CASE}

  fi

  JOB_NAME=step3

  CMD="perl ${PL_COUNT_KMER_ACROSS} -a ${KMER_CASE} -b ${KMER_CONTROL} -l ${PHENO_FILE} -c ${CUTOFF_FREQ} -d ${FNA_DIR} -o ${OUT_STEP3_FAS}"
  echo ${CMD}

  if [ "${QSUB_ENV}" -eq 1 ]; then
    qsub -cwd -S /bin/bash -N ${JOB_NAME} -l s_vmem=32G -l mem_req=32G  -e /dev/null -o /dev/null <<< "
      ${CMD}
    "
  else
    eval ${CMD}
    if [ $? -ne 0 ]; then
      echo_fail "Error: ${CMD}"
```

```
    fi
  fi
  wait_until_finish ${JOB_NAME}
fi

if [ ! -s "${OUT_STEP3_FAS}" ]; then
  echo_fail "Error: ${OUT_STEP3_FAS} is empty after STEP${STEP}"
elif [ ! -s "${OUT_STEP3_VAR}" ]; then
  echo_fail "Error: ${OUT_STEP3_VAR} is empty after STEP${STEP}"
fi

##############################################################
# calculate the relatedness matrix
##############################################################
STEP=4
disp_punctuate ${STEP}

SNP_MATRIX_FILE=`echo ${CORE_GENOME_FAS} | perl -pe 's/¥.f[a-z]+$/_noNa2SNP.matrix/g'`

if [ -s "${SNP_MATRIX_FILE}" ]; then

  echo "${SNP_MATRIX_FILE} already exists. Skipped."

else

  JOB_NAME=SNPcall

  CMD="R --vanilla --quiet < ${R_SNP_CALL} --args ${CORE_GENOME_FAS}"
  echo ${CMD}

  if [ "${QSUB_ENV}" -eq 1 ]; then
    qsub -cwd -S /bin/bash -N ${JOB_NAME} -l s_vmem=64G -l mem_req=64G  -e /dev/null -o /dev/null <<< "
      ${CMD}
    "
  else
    eval ${CMD}
    if [ $? -ne 0 ]; then
      echo_fail "Error: ${CMD}"
    fi
  fi
  wait_until_finish ${JOB_NAME}

  if [ ! -s "${SNP_MATRIX_FILE}" ]; then
    echo_fail "Error: ${SNP_MATRIX_FILE} is empty at STEP${STEP}"
  fi
fi


# ped, map => .bed
# matrix   => .linLoc.geno
if [ -s "${OUT_PREFIX}.cXX.txt" ]; then

  echo "${OUT_PREFIX}.cXX.txt already exists.  Skipped."

else

  CMD="perl ${PL_CONVERT_SNP2PLINK} -f ${SNP_MATRIX_FILE} -l ${PHENO_FILE}"
  echo ${CMD}
  eval ${CMD}
  if [ $? -ne 0 ]; then
    echo_fail "Error: ${CMD} "
  fi

  CMD="plink --file ${SNP_MATRIX_FILE} "
  CMD=${CMD}" --compound-genotypes --1 --map3 --noweb "
  CMD=${CMD}" --make-bed --out ${SNP_MATRIX_FILE}"
  echo ${CMD}
  eval ${CMD}
  if [ $? -ne 0 ]; then
    echo_fail "Error: ${CMD} "
  fi


  # binary ped => relatedness
  CMD="gemma -bfile ${SNP_MATRIX_FILE} -gk 1 -outdir . -o ${OUT_PREFIX}"
  echo ${CMD}
  eval ${CMD}
  if [ $? -ne 0 ]; then
    echo_fail "Error: ${CMD} "
  fi

  if [ ! -s "${OUT_PREFIX}.cXX.txt" ]; then
    echo_fail "Error: ${OUT_PREFIX}.cXX.txt is empyt after STEP${STEP}"
  fi
fi
```

```
#################################################################
# bugwas
#################################################################
STEP=5 # bugwas
disp_punctuate ${STEP}

PHENO_LIN_LOC_FILE=${PHENO_FILE}
GEN_LIN_LOC=${SNP_MATRIX_FILE}.linLoc.geno
RELATEDNESS_FILE=${OUT_PREFIX}.cXX.txt

if [ -s "${OUT_PREFIX}_var_lmmout_allSNPs_merged.txt" ]; then

  echo "${OUT_PREFIX}_var_lmmout_allSNPs_merged.txt already exists.  STEP${STEP} was skipped."

else

RSCRIPT_BUGWAS=${OUT_PREFIX}_exec_lin_loc.R
cat > ${RSCRIPT_BUGWAS} << EOF

  library(bugwas)

  gem.path="~/bin/gemma"

  output.dir="./"

  lin_loc(
    gen="${GEN_LIN_LOC}"
   ,pheno="${PHENO_LIN_LOC_FILE}"
   ,phylo="${TREE_FILE}"
   ,prefix="${OUT_PREFIX}"
   ,gem.path=gem.path
   ,var.matrix="${OUT_STEP3_VAR}"
   ,relmatrix="${RELATEDNESS_FILE}"
   ,output.dir=output.dir
  )

EOF

  CMD="R CMD BATCH ${RSCRIPT_BUGWAS}"
  echo ${CMD}
  eval ${CMD}
  if [ $? -ne 0 ]; then
    echo_fail "Error: ${CMD} "
  fi

fi



#################################################################
# annotation
#################################################################
STEP=6
disp_punctuate ${STEP}

FILE_TO_BE_ANNOTATED=""

if [ -s "${OUT_PREFIX}_var_lmmout_allSNPs_merged.txt" ]; then
  FILE_TO_BE_ANNOTATED="${OUT_PREFIX}_var_lmmout_allSNPs_merged.txt"
else
  FILE_TO_BE_ANNOTATED="${OUT_PREFIX}_var_lmmout_allSNPs.txt"
fi

CMD="perl ${PL_ANNOTATE} -d ${ANNOTATION_DIR} -f ${FILE_TO_BE_ANNOTATED}"
echo ${CMD}
eval ${CMD}
if [ $? -ne 0 ]; then
  echo_fail "Error: ${CMD} "
fi
```

Supplementary figure legends

Figure S1. Phylogenetic incongruence of the adhesin gene.

Left; The clonal phylogeny in Figure 1. Right; NJ-tree of the conserved portions (from nucleotide positions 544) of the adhesion gene. Notable incongruent tips are connected by the lines. Red and pink indicate carbapenem-resistant strains used for GWAS discovery and validation, respectively.

Figure S2. Maximum-likelihood phylogeny of strains in the original dataset and the carbapenem-susceptible ATCC 17978 strain.

The columns "STs" and "strains" between the tree and heatmap indicate sequence type and name of each strain. STs that were not determined by perfect matching of the alleles are kept blank. Green in the 1st, 2nd, 3rd columns of the heatmap indicates the presence of OXA-23, IS*Aba1* in the upstream of OXA-51, and OXA-51, respectively. Red and pink indicate carbapenem-resistant strains used for GWAS discovery and validation, respectively. The strains indicated by the dashed circle are probably *Acinetobacter* species that are different from *A. baumannii*.

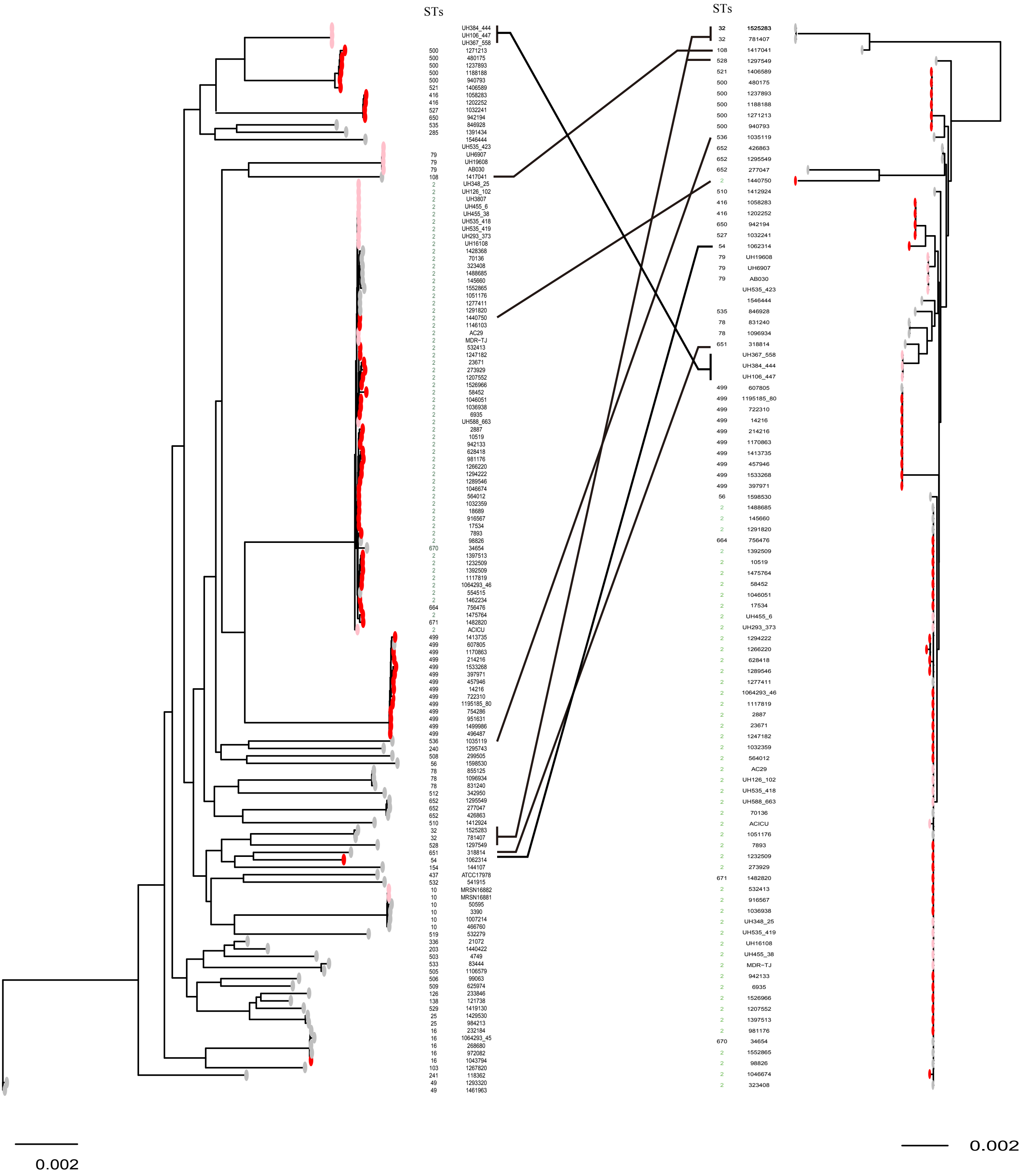
STs
UH384_444
UH106_447
UH367_558
500 1271213
500 480175
500 1237893
500 1188188
500 940793
521 1406589
416 1058283
416 1202252
527 1032241
650 942194
535 846928
285 1391434
1546444
UH535_423
79 UH6907
79 UH19608
79 AB030
108 1417041
2 UH348_25
2 UH126_102
2 UH3807
2 UH455_6
2 UH455_38
2 UH535_418
2 UH535_419
2 UH293_373
2 UH16108
2 1428368
2 70136
2 323408
2 1488685
2 145660
2 1552865
2 1051176
2 1277411
2 1291820
2 1440750
2 1146103
2 AC29
2 MDR−TJ
2 532413
2 1247182
2 23671
2 273929
2 1207552
2 1526966
2 58452
2 1046051
2 1036938
2 6935
2 UH588_663
2 2887
2 10519
2 942133
2 628418
2 981176
2 1266220
2 1294222
2 1289546
2 1046674
2 564012
2 1032359
2 18689
2 916567
2 17534
2 7893
2 98826
670 34654
2 1397513
2 1232509
2 1392509
2 1117819
2 1064293_46
2 554515
2 1462234
664 756476
2 1475764
671 1482820
2 ACICU
499 1413735
499 607805
499 1170863
499 214216
499 1533268
499 397971
499 457946
499 14216
499 722310
499 1195185_80
499 754286
499 951631
499 1499986
499 496487
536 1035119
240 1295743
508 299505
56 1598530
78 855125
78 1096934
78 831240
512 342950
652 1295549
652 277047
652 426863
510 1412924
32 1525283
32 781407
528 1297549
651 318814
54 1062314
154 144107
437 ATCC17978
532 541915
10 MRSN16882
10 MRSN16881
10 50595
10 3390
10 1007214
10 466760
519 532279
336 21072
203 1440422
503 4749
533 83444
505 1106579
506 99063
509 625974
126 233846
138 121738
529 1419130
25 1429530
25 984213
16 232184
16 1064293_45
16 268680
16 972082
16 1043794
103 1267820
241 118362
49 1293320
49 1461963
0.002
STs
32 1525283
32 781407
108 1417041
528 1297549
521 1406589
500 480175
500 1237893
500 1188188
500 1271213
500 940793
536 1035119
652 426863
652 1295549
652 277047
2 1440750
510 1412924
416 1058283
416 1202252
650 942194
527 1032241
54 1062314
79 UH19608
79 UH6907
79 AB030
UH535_423
1546444
535 846928
78 831240
78 1096934
651 318814
UH367_558
UH384_444
UH106_447
499 607805
499 1195185_80
499 722310
499 14216
499 214216
499 1170863
499 1413735
499 457946
499 1533268
499 397971
56 1598530
2 1488685
2 145660
2 1291820
664 756476
2 1392509
2 10519
2 1475764
2 58452
2 1046051
2 17534
2 UH455_6
2 UH293_373
2 1294222
2 1266220
2 628418
2 1289546
2 1277411
2 1064293_46
2 1117819
2 2887
2 23671
2 1247182
2 1032359
2 564012
2 AC29
2 UH126_102
2 UH535_418
2 UH588_663
2 70136
2 ACICU
2 1051176
2 7893
2 1232509
2 273929
671 1482820
2 532413
2 916567
2 1036938
2 UH348_25
2 UH535_419
2 UH16108
2 UH455_38
2 MDR−TJ
2 942133
2 6935
2 1526966
2 1207552
2 1397513
2 981176
670 34654
2 1552865
2 98826
2 1046674
2 323408
0.002

Figure S2

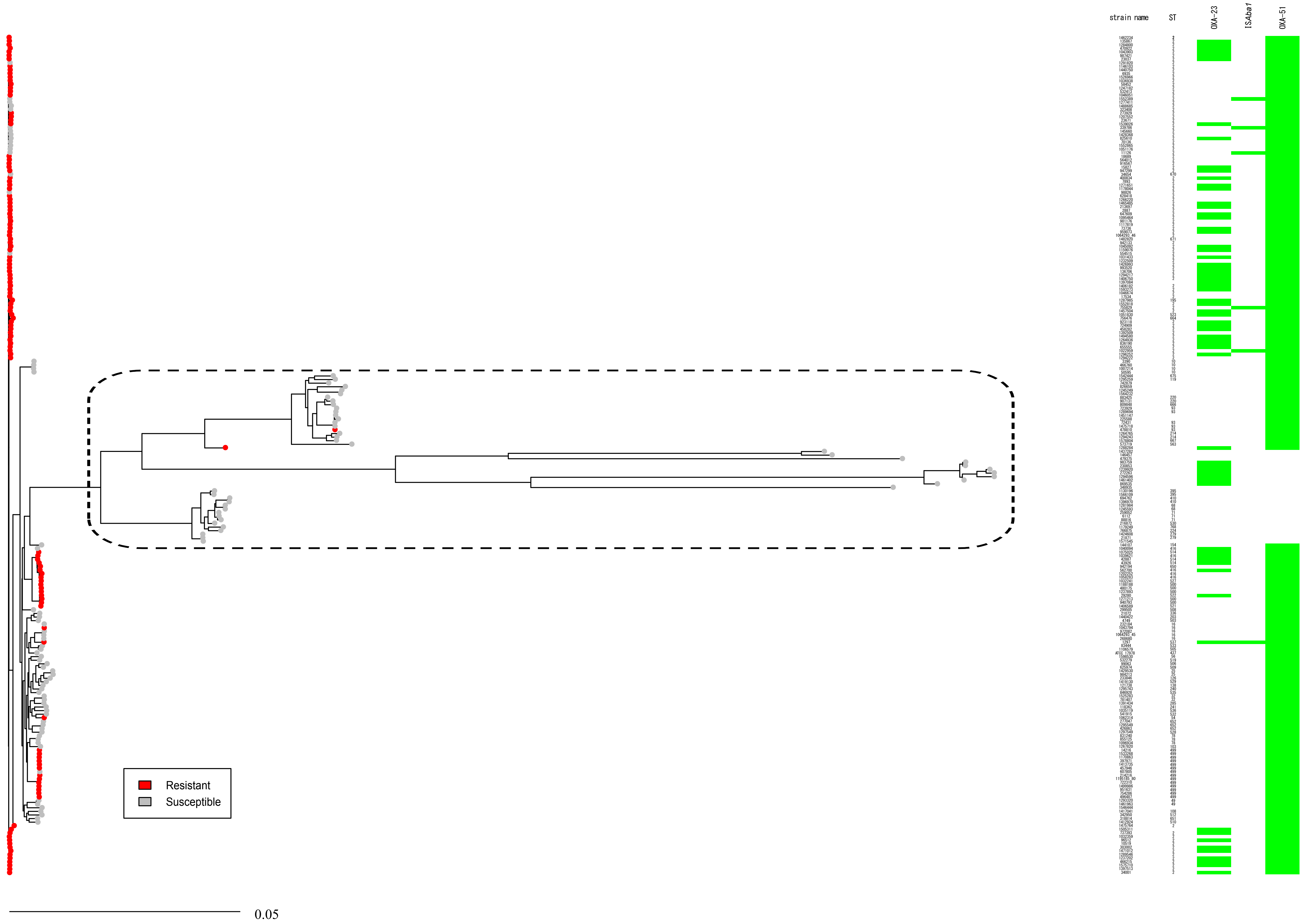
strain name
ST
OXA-23
ISAba1
OXA-51
Resistant
Susceptible
0.05

Table S1. List of the 212 kmers

kmers	increase in frequency	p-value	FDR p-value	mapped locus		mapped location	
						from	to
CATACGCACTACATCTAAACCATTACGAATG	70.5%	0.0000000341	0.0000003140	ABTJ_03740	alpha-hydroxyacid dehydrogenase, FMN-dependent L-lactate dehydrogenase	924	954
TACGCACTACATCTAAACCATTACGAATGCC	70.5%	0.0000000341	0.0000003140	ABTJ_03740	alpha-hydroxyacid dehydrogenase, FMN-dependent L-lactate dehydrogenase	922	952
ATACGCACTACATCTAAACCATTACGAATGC	70.5%	0.0000000341	0.0000003140	ABTJ_03740	alpha-hydroxyacid dehydrogenase, FMN-dependent L-lactate dehydrogenase	923	953
ACTTGATCTTTGTTATGAAGAAGACTCGAAC	70.5%	0.0000002550	0.0000016600	BL01_01510	rph, ribonuclease PH	516	546
TACTTGATCTTTGTTATGAAGAAGACTCGAA	70.5%	0.0000002550	0.0000016600	BL01_01510	rph, ribonuclease PH	515	545
ATTACTTGATCTTTGTTATGAAGAAGACTCG	70.5%	0.0000002550	0.0000016600	BL01_01510	rph, ribonuclease PH	513	543
AACAAAGATCAAGTAATACTTCATCTTGATA	70.5%	0.0000002550	0.0000016600	BL01_01510	rph, ribonuclease PH	499	529
ATAACAAAGATCAAGTAATACTTCATCTTGA	70.5%	0.0000002550	0.0000016600	BL01_01510	rph, ribonuclease PH	501	531
ATCAAGATGAAGTATTACTTGATCTTTGTTA	70.5%	0.0000002550	0.0000016600	BL01_01510	rph, ribonuclease PH	500	530
TCGAGTCTTCTTCATAACAAAGATCAAGTAA	70.5%	0.0000002550	0.0000016600	BL01_01510	rph, ribonuclease PH	514	544
ACAAAGATCAAGTAATACTTCATCTTGATAC	70.5%	0.0000002550	0.0000016600	BL01_01510	rph, ribonuclease PH	498	528
AAAGCTCAAACAGTTTACCGATGCCCGTATT	75.4%	0.0000004560	0.0000022900	ACICU_02301	Ethanolamine ammonia-lyase, small subunit	57	87
ACGGGCATCGGTAAACTGTTTGAGCTTTTCC	75.4%	0.0000004560	0.0000022900	ACICU_02301	Ethanolamine ammonia-lyase, small subunit	54	84
TACGGGCATCGGTAAACTGTTTGAGCTTTTC	75.4%	0.0000004560	0.0000022900	ACICU_02301	Ethanolamine ammonia-lyase, small subunit	55	85
AAAAGCTCAAACAGTTTACCGATGCCCGTAT	75.4%	0.0000004560	0.0000022900	ACICU_02301	Ethanolamine ammonia-lyase, small subunit	56	86
CGGCTTGTTCCCCAATCACAATCGGTTGAAC	73.8%	0.0000134000	0.0000648000	ACICU_02910	putative surface adhesion protein	1375	1405
AGTTCAACCGATTGTGATTGGGGAACAAGCC	73.8%	0.0000134000	0.0000648000	ACICU_02910	putative surface adhesion protein	1374	1404
TTCAACCGATTGTGATTGGGGAACAAGCCGC	73.8%	0.0000134000	0.0000648000	ACICU_02910	putative surface adhesion protein	1376	1406
ACGCTAAAATTGTAAGCTTGTGTGCAGCGTC	73.8%	0.0000138000	0.0000648000	ACICU_00262	Homoserine dehydrogenase	607	637
ACGCTGCACACAAGCTTACAATTTTAGCGTC	73.8%	0.0000138000	0.0000648000	ACICU_00262	Homoserine dehydrogenase	608	638
AGACGCTAAAATTGTAAGCTTGTGTGCAGCG	73.8%	0.0000138000	0.0000648000	ACICU_00262	Homoserine dehydrogenase	609	639
CGCTAAAATTGTAAGCTTGTGTGCAGCGTCA	73.8%	0.0000138000	0.0000648000	ACICU_00262	Homoserine dehydrogenase	606	636
TAGCTCAAGATGGGTTTTATGTGATTATTAA	72.1%	0.0000467000	0.0001606250	ACICU_01449	Dehydrogenase with different specificities	74	104
AACCCATCTTGAGCTAAGCGTTCTGCAATAG	72.1%	0.0000467000	0.0001606250	ACICU_01449	Dehydrogenase with different specificities	59	89
ATAATCACATAAAACCCATCTTGAGCTAAGC	72.1%	0.0000467000	0.0001606250	ACICU_01449	Dehydrogenase with different specificities	71	101
ACCCATCTTGAGCTAAGCGTTCTGCAATAGC	72.1%	0.0000467000	0.0001606250	ACICU_01449	Dehydrogenase with different specificities	58	88
AATCACATAAAACCCATCTTGAGCTAAGCGT	72.1%	0.0000467000	0.0001606250	ACICU_01449	Dehydrogenase with different specificities	69	99
TAATAATCACATAAAACCCATCTTGAGCTAA	72.1%	0.0000467000	0.0001606250	ACICU_01449	Dehydrogenase with different specificities	73	103
AATAATCACATAAAACCCATCTTGAGCTAAG	72.1%	0.0000467000	0.0001606250	ACICU_01449	Dehydrogenase with different specificities	72	102
AAACCCATCTTGAGCTAAGCGTTCTGCAATA	72.1%	0.0000467000	0.0001606250	ACICU_01449	Dehydrogenase with different specificities	60	90
CGCTTAGCTCAAGATGGGTTTTATGTGATTA	72.1%	0.0000467000	0.0001606250	ACICU_01449	Dehydrogenase with different specificities	70	100
CCCATCTTGAGCTAAGCGTTCTGCAATAGCA	72.1%	0.0000467000	0.0001606250	ACICU_01449	Dehydrogenase with different specificities	57	87
ATCGTAGTATTCAGCTCTGCTACACCCGTAA	72.1%	0.0001794940	0.0004412730	ACICU_02910	putative surface adhesion protein	3551	3581
ATCTTACGGGTGTAGCAGAGCTGAATACTAC	72.1%	0.0001794940	0.0004412730	ACICU_02910	putative surface adhesion protein	3548	3578
ATTCAGCTCTGCTACACCCGTAAGATATGAA	72.1%	0.0001794940	0.0004412730	ACICU_02910	putative surface adhesion protein	3543	3573
CAGCTCTGCTACACCCGTAAGATATGAACCA	72.1%	0.0001794940	0.0004412730	ACICU_02910	putative surface adhesion protein	3540	3570
TTCAGCTCTGCTACACCCGTAAGATATGAAC	72.1%	0.0001794940	0.0004412730	ACICU_02910	putative surface adhesion protein	3542	3572
CATATCTTACGGGTGTAGCAGAGCTGAATAC	72.1%	0.0001794940	0.0004412730	ACICU_02910	putative surface adhesion protein	3545	3575
CGTAGTATTCAGCTCTGCTACACCCGTAAGA	72.1%	0.0001794940	0.0004412730	ACICU_02910	putative surface adhesion protein	3549	3579
CGGGTGTAGCAGAGCTGAATACTACGATTCA	72.1%	0.0001794940	0.0004412730	ACICU_02910	putative surface adhesion protein	3554	3584
CTGAATCGTAGTATTCAGCTCTGCTACACCC	72.1%	0.0001794940	0.0004412730	ACICU_02910	putative surface adhesion protein	3555	3585
ATATCTTACGGGTGTAGCAGAGCTGAATACT	72.1%	0.0001794940	0.0004412730	ACICU_02910	putative surface adhesion protein	3546	3576
AATCGTAGTATTCAGCTCTGCTACACCCGTA	72.1%	0.0001794940	0.0004412730	ACICU_02910	putative surface adhesion protein	3552	3582
ACGGGTGTAGCAGAGCTGAATACTACGATTC	72.1%	0.0001794940	0.0004412730	ACICU_02910	putative surface adhesion protein	3553	3583
TATTCAGCTCTGCTACACCCGTAAGATATGA	72.1%	0.0001794940	0.0004412730	ACICU_02910	putative surface adhesion protein	3544	3574
TCAGCTCTGCTACACCCGTAAGATATGAACC	72.1%	0.0001794940	0.0004412730	ACICU_02910	putative surface adhesion protein	3541	3571
GATGGTTCATATCTTACGGGTGTAGCAGAGC	72.1%	0.0001794940	0.0004412730	ACICU_02910	putative surface adhesion protein	3538	3568
CTTACGGGTGTAGCAGAGCTGAATACTACGA	72.1%	0.0001794940	0.0004412730	ACICU_02910	putative surface adhesion protein	3550	3580
AGATGGTTCATATCTTACGGGTGTAGCAGAG	72.1%	0.0001794940	0.0004412730	ACICU_02910	putative surface adhesion protein	3537	3567
ATGGTTCATATCTTACGGGTGTAGCAGAGCT	72.1%	0.0001794940	0.0004412730	ACICU_02910	putative surface adhesion protein	3539	3569
TATCTTACGGGTGTAGCAGAGCTGAATACTA	72.1%	0.0001794940	0.0004412730	ACICU_02910	putative surface adhesion protein	3547	3577
GTCAATACCTTCAACGTCAAAAGTTGGGTCC	72.1%	0.0001861630	0.0004412730	ACICU_00262	Homoserine dehydrogenase	579	609
TAAATGCGCCACTTGACGATATTGCACCGAA	70.5%	0.0003957370	0.0007104180	ACICU_02437	putative surface adhesion protein	3743	3773
ATTTCAATAAATGCGCCACTTGACGATATTG	70.5%	0.0003957370	0.0007104180	ACICU_02437	putative surface adhesion protein	3736	3766
TTTCAATAAATGCGCCACTTGACGATATTGC	70.5%	0.0003957370	0.0007104180	ACICU_02437	putative surface adhesion protein	3737	3767
ATATCGTCAAGTGGCGCATTTATTGAAATAG	70.5%	0.0003957370	0.0007104180	ACICU_02437	putative surface adhesion protein	3734	3764
AATATCGTCAAGTGGCGCATTTATTGAAATA	70.5%	0.0003957370	0.0007104180	ACICU_02910	putative surface adhesion protein	3735	3765
TATCGTCAAGTGGCGCATTTATTGAAATAGC	70.5%	0.0003957370	0.0007104180	ACICU_02910	putative surface adhesion protein	3733	3763
AATGCGCCACTTGACGATATTGCACCGAATC	70.5%	0.0003957370	0.0007104180	ACICU_02910	putative surface adhesion protein	3745	3775
AAATGCGCCACTTGACGATATTGCACCGAAT	70.5%	0.0003957370	0.0007104180	ACICU_02910	putative surface adhesion protein	3744	3774
CAATAAATGCGCCACTTGACGATATTGCACC	70.5%	0.0003957370	0.0007104180	ACICU_02910	putative surface adhesion protein	3740	3770
TGCGCCACTTGACGATATTGCACCGAATCCA	70.5%	0.0003957370	0.0007104180	ACICU_02910	putative surface adhesion protein	3747	3777
ATGCGCCACTTGACGATATTGCACCGAATCC	70.5%	0.0003957370	0.0007104180	ACICU_02910	putative surface adhesion protein	3746	3776
TCAATAAATGCGCCACTTGACGATATTGCAC	70.5%	0.0003957370	0.0007104180	ACICU_02910	putative surface adhesion protein	3739	3769
TTCAATAAATGCGCCACTTGACGATATTGCA	70.5%	0.0003957370	0.0007104180	ACICU_02910	putative surface adhesion protein	3738	3768
AATAAATGCGCCACTTGACGATATTGCACCG	70.5%	0.0003957370	0.0007104180	ACICU_02910	putative surface adhesion protein	3741	3771
ATAAATGCGCCACTTGACGATATTGCACCGA	70.5%	0.0003957370	0.0007104180	ACICU_02910	putative surface adhesion protein	3742	3772
CCAGCATAGTTCACTTTAGTGACTGCCTGCG	70.5%	0.0004142740	0.0007104180	ACICU_02437	Gamma-aminobutyrate permease	965	995
CAGCATAGTTCACTTTAGTGACTGCCTGCGG	70.5%	0.0004142740	0.0007104180	ACICU_02437	Gamma-aminobutyrate permease	964	994
ACCGCAGGCAGTCACTAAAGTGAACTATGCT	70.5%	0.0004142740	0.0007104180	ACICU_02437	Gamma-aminobutyrate permease	963	993
ACCAGCATAGTTCACTTTAGTGACTGCCTGC	70.5%	0.0004142740	0.0007104180	ACICU_02437	Gamma-aminobutyrate permease	966	996
TCACTAAAGTGAACTATGCTGGTGTACCAGC	72.1%	0.0004166840	0.0007104180	ACICU_02437	Gamma-aminobutyrate permease	974	1004
CTGGTACACCAGCATAGTTCACTTTAGTGAC	72.1%	0.0004166840	0.0007104180	ACICU_02437	Gamma-aminobutyrate permease	973	1003
CACTAAAGTGAACTATGCTGGTGTACCAGCA	72.1%	0.0004166840	0.0007104180	ACICU_02437	Gamma-aminobutyrate permease	975	1005
AGTCACTAAAGTGAACTATGCTGGTGTACCA	72.1%	0.0004166840	0.0007104180	ACICU_02437	Gamma-aminobutyrate permease	972	1002
CAATCGCGCTGGTTGGGGATATGTTGATAGC	70.5%	0.0004178080	0.0007104180	ACICU_02910	putative surface adhesion protein	6717	6747
TATCAACATATCCCCAACCAGCGCGATTGCC	70.5%	0.0004178080	0.0007104180	ACICU_02910	putative surface adhesion protein	6715	6745
AATCGCGCTGGTTGGGGATATGTTGATAGCC	70.5%	0.0004178080	0.0007104180	ACICU_02437	putative surface adhesion protein	6718	6748
ATCGCGCTGGTTGGGGATATGTTGATAGCCA	70.5%	0.0004178080	0.0007104180	ACICU_02437	putative surface adhesion protein	6719	6749
TCGCGCTGGTTGGGGATATGTTGATAGCCAA	70.5%	0.0004178080	0.0007104180	ACICU_02437	putative surface adhesion protein	6720	6750
CTATCAACATATCCCCAACCAGCGCGATTGC	70.5%	0.0004178080	0.0007104180	ACICU_02437	putative surface adhesion protein	6716	6746
TCACAAGCAGATGAATTAGAACAAGCCGTGA	70.5%	0.0006782410	0.0010507360	ACICU_01307	ATPase with chaperone activity, ATP-binding subunit	496	526
GAACAAGCCGTGACTCCAACTGCAAAAACTC	70.5%	0.0006782410	0.0010507360	ACICU_01307	ATPase with chaperone activity, ATP-binding subunit	514	544
CAAGCCGTGACTCCAACTGCAAAAACTCAAA	70.5%	0.0006782410	0.0010507360	ACICU_01307	ATPase with chaperone activity, ATP-binding subunit	517	547
TTGCAGTTGGAGTCACGGCTTGTTCTAATTC	70.5%	0.0006782410	0.0010507360	ACICU_01307	ATPase with chaperone activity, ATP-binding subunit	508	538
AGCAGATGAATTAGAACAAGCCGTGACTCCA	70.5%	0.0006782410	0.0010507360	ACICU_01307	ATPase with chaperone activity, ATP-binding subunit	501	531
TAGAACAAGCCGTGACTCCAACTGCAAAAAC	70.5%	0.0006782410	0.0010507360	ACICU_01307	ATPase with chaperone activity, ATP-binding subunit	512	542
AAGCCGTGACTCCAACTGCAAAAACTCAAAA	70.5%	0.0006782410	0.0010507360	ACICU_01307	ATPase with chaperone activity, ATP-binding subunit	518	548
CCGTGACTCCAACTGCAAAAACTCAAAAAAC	70.5%	0.0006782410	0.0010507360	ACICU_01307	ATPase with chaperone activity, ATP-binding subunit	521	551
AATTAGAACAAGCCGTGACTCCAACTGCAAA	70.5%	0.0006782410	0.0010507360	ACICU_01307	ATPase with chaperone activity, ATP-binding subunit	509	539
AGCCGTGACTCCAACTGCAAAAACTCAAAAA	70.5%	0.0006782410	0.0010507360	ACICU_01307	ATPase with chaperone activity, ATP-binding subunit	519	549
TGAATTAGAACAAGCCGTGACTCCAACTGCA	70.5%	0.0006782410	0.0010507360	ACICU_01307	ATPase with chaperone activity, ATP-binding subunit	507	537
AACAAGCCGTGACTCCAACTGCAAAAACTCA	70.5%	0.0006782410	0.0010507360	ACICU_01307	ATPase with chaperone activity, ATP-binding subunit	515	545
CAGATGAATTAGAACAAGCCGTGACTCCAAC	70.5%	0.0006782410	0.0010507360	ACICU_01307	ATPase with chaperone activity, ATP-binding subunit	503	533
TTGGAGTCACGGCTTGTTCTAATTCATCTGC	70.5%	0.0006782410	0.0010507360	ACICU_01307	ATPase with chaperone activity, ATP-binding subunit	502	532
CAAGCAGATGAATTAGAACAAGCCGTGACTC	70.5%	0.0006782410	0.0010507360	ACICU_01307	ATPase with chaperone activity, ATP-binding subunit	499	529
AGATGAATTAGAACAAGCCGTGACTCCAACT	70.5%	0.0006782410	0.0010507360	ACICU_01307	ATPase with chaperone activity, ATP-binding subunit	504	534
ACAAGCCGTGACTCCAACTGCAAAAACTCAA	70.5%	0.0006782410	0.0010507360	ACICU_01307	ATPase with chaperone activity, ATP-binding subunit	516	546
CACAAGCAGATGAATTAGAACAAGCCGTGAC	70.5%	0.0006782410	0.0010507360	ACICU_01307	ATPase with chaperone activity, ATP-binding subunit	497	527
TTTTTTGAGTTTTTGCAGTTGGAGTCACGGC	70.5%	0.0006782410	0.0010507360	ACICU_01307	ATPase with chaperone activity, ATP-binding subunit	520	550
ATTAGAACAAGCCGTGACTCCAACTGCAAAA	70.5%	0.0006782410	0.0010507360	ACICU_01307	ATPase with chaperone activity, ATP-binding subunit	510	540
AAGCAGATGAATTAGAACAAGCCGTGACTCC	70.5%	0.0006782410	0.0010507360	ACICU_01307	ATPase with chaperone activity, ATP-binding subunit	500	530
CAGTTGGAGTCACGGCTTGTTCTAATTCATC	70.5%	0.0006782410	0.0010507360	ACICU_01307	ATPase with chaperone activity, ATP-binding subunit	505	535
ACAAGCAGATGAATTAGAACAAGCCGTGACT	70.5%	0.0006782410	0.0010507360	ACICU_01307	ATPase with chaperone activity, ATP-binding subunit	498	528
ATGAATTAGAACAAGCCGTGACTCCAACTGC	70.5%	0.0006782410	0.0010507360	ACICU_01307	ATPase with chaperone activity, ATP-binding subunit	506	536
CACGGCTTGTTCTAATTCATCTGCTTGTGAA	70.5%	0.0006782410	0.0010507360	ACICU_01307	ATPase with chaperone activity, ATP-binding subunit	495	525
TTAGAACAAGCCGTGACTCCAACTGCAAAAA	70.5%	0.0006782410	0.0010507360	ACICU_01307	ATPase with chaperone activity, ATP-binding subunit	511	541
AGAACAAGCCGTGACTCCAACTGCAAAAACT	70.5%	0.0006782410	0.0010507360	ACICU_01307	ATPase with chaperone activity, ATP-binding subunit	513	543
AGCCGGTGAGTAATCGAGCTGACCTAACTGA	70.5%	0.0008232960	0.0011782070	ACICU_02439	Adenosylmethionine-8-amino-7-oxononanoate aminotransferase	270	300

AAGAAAGCGGATGACCAAATTGGAAAGCCGG	70.5%	0.0008232960	0.0011782070	ACICU_02439	Adenosylmethionine-8-amino-7-oxononanoate aminotransferase	295	325
AAGCCGGTGAGTAATCGAGCTGACCTAACTG	70.5%	0.0008232960	0.0011782070	ACICU_02439	Adenosylmethionine-8-amino-7-oxononanoate aminotransferase	271	301
TTAGGTCAGCTCGATTACTCACCGGCTTTCC	70.5%	0.0008232960	0.0011782070	ACICU_02439	Adenosylmethionine-8-amino-7-oxononanoate aminotransferase	274	304
CGGATGACCAAATTGGAAAGCCGGTGAGTAA	70.5%	0.0008232960	0.0011782070	ACICU_02439	Adenosylmethionine-8-amino-7-oxononanoate aminotransferase	288	318
ATTGGAAAGCCGGTGAGTAATCGAGCTGACC	70.5%	0.0008232960	0.0011782070	ACICU_02439	Adenosylmethionine-8-amino-7-oxononanoate aminotransferase	277	307
GAAAGCCGGTGAGTAATCGAGCTGACCTAAC	70.5%	0.0008232960	0.0011782070	ACICU_02439	Adenosylmethionine-8-amino-7-oxononanoate aminotransferase	273	303
CAAATTGGAAAGCCGGTGAGTAATCGAGCTG	70.5%	0.0008232960	0.0011782070	ACICU_02439	Adenosylmethionine-8-amino-7-oxononanoate aminotransferase	280	310
AAAGCCGGTGAGTAATCGAGCTGACCTAACT	70.5%	0.0008232960	0.0011782070	ACICU_02439	Adenosylmethionine-8-amino-7-oxononanoate aminotransferase	272	302
AAAGCGGATGACCAAATTGGAAAGCCGGTGA	70.5%	0.0008232960	0.0011782070	ACICU_02439	Adenosylmethionine-8-amino-7-oxononanoate aminotransferase	292	322
AAAGAAAGCGGATGACCAAATTGGAAAGCCG	70.5%	0.0008232960	0.0011782070	ACICU_02439	Adenosylmethionine-8-amino-7-oxononanoate aminotransferase	296	326
AAGCGGATGACCAAATTGGAAAGCCGGTGAG	70.5%	0.0008232960	0.0011782070	ACICU_02439	Adenosylmethionine-8-amino-7-oxononanoate aminotransferase	291	321
TCGATTACTCACCGGCTTTCCAATTTGGTCA	70.5%	0.0008232960	0.0011782070	ACICU_02439	Adenosylmethionine-8-amino-7-oxononanoate aminotransferase	284	314
AAAAGAAAGCGGATGACCAAATTGGAAAGCC	70.5%	0.0008232960	0.0011782070	ACICU_02439	Adenosylmethionine-8-amino-7-oxononanoate aminotransferase	297	327
AAATTGGAAAGCCGGTGAGTAATCGAGCTGA	70.5%	0.0008232960	0.0011782070	ACICU_02439	Adenosylmethionine-8-amino-7-oxononanoate aminotransferase	279	309
TACTCACCGGCTTTCCAATTTGGTCATCCGC	70.5%	0.0008232960	0.0011782070	ACICU_02439	Adenosylmethionine-8-amino-7-oxononanoate aminotransferase	289	319
ACCAAATTGGAAAGCCGGTGAGTAATCGAGC	70.5%	0.0008232960	0.0011782070	ACICU_02439	Adenosylmethionine-8-amino-7-oxononanoate aminotransferase	282	312
ATGACCAAATTGGAAAGCCGGTGAGTAATCG	70.5%	0.0008232960	0.0011782070	ACICU_02439	Adenosylmethionine-8-amino-7-oxononanoate aminotransferase	285	315
AGGTCAGCTCGATTACTCACCGGCTTTCCAA	70.5%	0.0008232960	0.0011782070	ACICU_02439	Adenosylmethionine-8-amino-7-oxononanoate aminotransferase	276	306
CACCGGCTTTCCAATTTGGTCATCCGCTTTC	70.5%	0.0008232960	0.0011782070	ACICU_02439	Adenosylmethionine-8-amino-7-oxononanoate aminotransferase	293	323
AATTGGAAAGCCGGTGAGTAATCGAGCTGAC	70.5%	0.0008232960	0.0011782070	ACICU_02439	Adenosylmethionine-8-amino-7-oxononanoate aminotransferase	278	308
CTCGATTACTCACCGGCTTTCCAATTTGGTC	70.5%	0.0008232960	0.0011782070	ACICU_02439	Adenosylmethionine-8-amino-7-oxononanoate aminotransferase	283	313
ACCGGCTTTCCAATTTGGTCATCCGCTTTCT	70.5%	0.0008232960	0.0011782070	ACICU_02439	Adenosylmethionine-8-amino-7-oxononanoate aminotransferase	294	324
ACTCACCGGCTTTCCAATTTGGTCATCCGCT	70.5%	0.0008232960	0.0011782070	ACICU_02439	Adenosylmethionine-8-amino-7-oxononanoate aminotransferase	290	320
AGCTCGATTACTCACCGGCTTTCCAATTTGG	70.5%	0.0008232960	0.0011782070	ACICU_02439	Adenosylmethionine-8-amino-7-oxononanoate aminotransferase	281	311
ATACACCACCAGACTCACCACCAATCCAATA	70.5%	0.0009531100	0.0012919930	ACICU_01366	Purine-cytosine permease	1311	1341
ACTCACCACCAATCCAATAACCCATGAAATA	70.5%	0.0009531100	0.0012919930	ACICU_01366	Purine-cytosine permease	1299	1329
CAGACTCACCACCAATCCAATAACCCATGAA	70.5%	0.0009531100	0.0012919930	ACICU_01366	Purine-cytosine permease	1302	1332
ATGGGTTATTGGATTGGTGGTGAGTCTGGTG	70.5%	0.0009531100	0.0012919930	ACICU_01366	Purine-cytosine permease	1305	1335
CCAGACTCACCACCAATCCAATAACCCATGA	70.5%	0.0009531100	0.0012919930	ACICU_01366	Purine-cytosine permease	1303	1333
CACCACCAGACTCACCACCAATCCAATAACC	70.5%	0.0009531100	0.0012919930	ACICU_01366	Purine-cytosine permease	1308	1338
ACCACCAGACTCACCACCAATCCAATAACCC	70.5%	0.0009531100	0.0012919930	ACICU_01366	Purine-cytosine permease	1307	1337
TACACCACCAGACTCACCACCAATCCAATAA	70.5%	0.0009531100	0.0012919930	ACICU_01366	Purine-cytosine permease	1310	1340
ATATTTCATGGGTTATTGGATTGGTGGTGAG	70.5%	0.0009531100	0.0012919930	ACICU_01366	Purine-cytosine permease	1298	1328
ATTTCATGGGTTATTGGATTGGTGGTGAGTC	70.5%	0.0009531100	0.0012919930	ACICU_01366	Purine-cytosine permease	1300	1330
CCACCAGACTCACCACCAATCCAATAACCCA	70.5%	0.0009531100	0.0012919930	ACICU_01366	Purine-cytosine permease	1306	1336
AGACTCACCACCAATCCAATAACCCATGAAA	70.5%	0.0009531100	0.0012919930	ACICU_01366	Purine-cytosine permease	1301	1331
ACACCACCAGACTCACCACCAATCCAATAAC	70.5%	0.0009531100	0.0012919930	ACICU_01366	Purine-cytosine permease	1309	1339
ACCAGACTCACCACCAATCCAATAACCCATG	70.5%	0.0009531100	0.0012919930	ACICU_01366	Purine-cytosine permease	1304	1334
TCACCACCAATCCAATAACCCATGAAATATC	70.5%	0.0009531100	0.0012919930	ACICU_01366	Purine-cytosine permease	1297	1327
CCTGAATCGTAGTATTCAGCTCTGCTACACC	70.5%	0.0010469690	0.0012934710	ACICU_02910	putative surface adhesion protein	3556	3586
CCAGTAATTTAGTTTTCTCAGAAGATGGTTC	70.5%	0.0010469690	0.0012934710	ACICU_02910	putative surface adhesion protein	3515	3545
CAAAAACCTGAATCGTAGTATTCAGCTCTGC	70.5%	0.0010469690	0.0012934710	ACICU_02910	putative surface adhesion protein	3562	3592
AAAAACCTGAATCGTAGTATTCAGCTCTGCT	70.5%	0.0010469690	0.0012934710	ACICU_02910	putative surface adhesion protein	3561	3591
TATGAACCATCTTCTGAGAAAACTAAATTAC	70.5%	0.0010469690	0.0012934710	ACICU_02910	putative surface adhesion protein	3518	3548
AAAACCTGAATCGTAGTATTCAGCTCTGCTA	70.5%	0.0010469690	0.0012934710	ACICU_02910	putative surface adhesion protein	3560	3590
ACCATCTTCTGAGAAAACTAAATTACTGGCT	70.5%	0.0010469690	0.0012934710	ACICU_02910	putative surface adhesion protein	3513	3543
GATCAAAAACCTGAATCGTAGTATTCAGCTC	70.5%	0.0010469690	0.0012934710	ACICU_02910	putative surface adhesion protein	3565	3595
AACCATCTTCTGAGAAAACTAAATTACTGGC	70.5%	0.0010469690	0.0012934710	ACICU_02910	putative surface adhesion protein	3514	3544
CATCTTCTGAGAAAACTAAATTACTGGCTGG	70.5%	0.0010469690	0.0012934710	ACICU_02910	putative surface adhesion protein	3511	3541
CAGCCAGTAATTTAGTTTTCTCAGAAGATGG	70.5%	0.0010469690	0.0012934710	ACICU_02910	putative surface adhesion protein	3512	3542
AACCTGAATCGTAGTATTCAGCTCTGCTACA	70.5%	0.0010469690	0.0012934710	ACICU_02910	putative surface adhesion protein	3558	3588
CAGAGCTGAATACTACGATTCAGGTTTTTGA	70.5%	0.0010469690	0.0012934710	ACICU_02910	putative surface adhesion protein	3563	3593
ATGAACCATCTTCTGAGAAAACTAAATTACT	70.5%	0.0010469690	0.0012934710	ACICU_02910	putative surface adhesion protein	3517	3547
ACCTGAATCGTAGTATTCAGCTCTGCTACAC	70.5%	0.0010469690	0.0012934710	ACICU_02910	putative surface adhesion protein	3557	3587
AAACCTGAATCGTAGTATTCAGCTCTGCTAC	70.5%	0.0010469690	0.0012934710	ACICU_02910	putative surface adhesion protein	3559	3589
ATCAAAAACCTGAATCGTAGTATTCAGCTCT	70.5%	0.0010469690	0.0012934710	ACICU_02910	putative surface adhesion protein	3564	3594
ATGATCAAAAACCTGAATCGTAGTATTCAGC	70.5%	0.0010469690	0.0012934710	ACICU_02910	putative surface adhesion protein	3567	3597
CAGTAATTTAGTTTTCTCAGAAGATGGTTCA	70.5%	0.0010469690	0.0012934710	ACICU_02910	putative surface adhesion protein	3516	3546
AGCTGAATACTACGATTCAGGTTTTTGATCA	70.5%	0.0010469690	0.0012934710	ACICU_02910	putative surface adhesion protein	3566	3596
ATATGAACCATCTTCTGAGAAAACTAAATTA	70.5%	0.0010469690	0.0012934710	ACICU_02910	putative surface adhesion protein	3519	3549
TAAGCGTTATGCGCTACAGGCCAATATTCAA	70.5%	0.0011960860	0.0014739650	ACICU_00264	Site-specific recombinase XerD	708	738
AATTGTACACTTGTGGAAGGTGTAGAAAAAA	70.5%	0.0012448470	0.0014852940	ACICU_00272	predicted phosphohydrolase	635	665
AAATTGTACACTTGTGGAAGGTGTAGAAAAA	70.5%	0.0012448470	0.0014852940	ACICU_00272	predicted phosphohydrolase	636	666
AACGGCTTAAATTGTACACTTGTGGAAGGTG	70.5%	0.0012448470	0.0014852940	ACICU_00272	predicted phosphohydrolase	644	674
AAACGGCTTAAATTGTACACTTGTGGAAGGT	70.5%	0.0012448470	0.0014852940	ACICU_00272	predicted phosphohydrolase	645	675
ATTGTACACTTGTGGAAGGTGTAGAAAAAAA	70.5%	0.0012448470	0.0014852940	ACICU_00272	predicted phosphohydrolase	634	664
TTGTACACTTGTGGAAGGTGTAGAAAAAAAC	70.5%	0.0012448470	0.0014852940	ACICU_00272	predicted phosphohydrolase	633	663
CGGCTTAAATTGTACACTTGTGGAAGGTGTA	70.5%	0.0012448470	0.0014852940	ACICU_00272	predicted phosphohydrolase	642	672
ACACCTTCCACAAGTGTACAATTTAAGCCGT	70.5%	0.0012448470	0.0014852940	ACICU_00272	predicted phosphohydrolase	643	673
ACTTTAAAGCTAGTTCAACCGATTGTGATTG	70.5%	0.0013420710	0.0015446480	ACICU_02910	putative surface adhesion protein	1363	1393
CCCAATCACAATCGGTTGAACTAGCTTTAAA	70.5%	0.0013420710	0.0015446480	ACICU_02910	putative surface adhesion protein	1365	1395
ATCACAATCGGTTGAACTAGCTTTAAAGTAA	70.5%	0.0013420710	0.0015446480	ACICU_02910	putative surface adhesion protein	1361	1391
TAGTTCAACCGATTGTGATTGGGGAACAAGC	70.5%	0.0013420710	0.0015446480	ACICU_02910	putative surface adhesion protein	1373	1403
CCCCAATCACAATCGGTTGAACTAGCTTTAA	70.5%	0.0013420710	0.0015446480	ACICU_02910	putative surface adhesion protein	1366	1396
CTAGTTCAACCGATTGTGATTGGGGAACAAG	70.5%	0.0013420710	0.0015446480	ACICU_02910	putative surface adhesion protein	1372	1402
CCAATCACAATCGGTTGAACTAGCTTTAAAG	70.5%	0.0013420710	0.0015446480	ACICU_02910	putative surface adhesion protein	1364	1394
AATCACAATCGGTTGAACTAGCTTTAAAGTA	70.5%	0.0013420710	0.0015446480	ACICU_02910	putative surface adhesion protein	1362	1392
TTGTTCCCCAATCACAATCGGTTGAACTAGC	70.5%	0.0013420710	0.0015446480	ACICU_02910	putative surface adhesion protein	1371	1401
AGCTAGTTCAACCGATTGTGATTGGGGAACA	70.5%	0.0013420710	0.0015446480	ACICU_02910	putative surface adhesion protein	1370	1400
AAGCTAGTTCAACCGATTGTGATTGGGGAAC	70.5%	0.0013420710	0.0015446480	ACICU_02910	putative surface adhesion protein	1369	1399
TCACAATCGGTTGAACTAGCTTTAAAGTAAA	70.5%	0.0013420710	0.0015446480	ACICU_02910	putative surface adhesion protein	1360	1390
AAAGCTAGTTCAACCGATTGTGATTGGGGAA	70.5%	0.0013420710	0.0015446480	ACICU_02910	putative surface adhesion protein	1368	1398
TAAAGCTAGTTCAACCGATTGTGATTGGGGA	70.5%	0.0013420710	0.0015446480	ACICU_02910	putative surface adhesion protein	1367	1397
CACAATCGGTTGAACTAGCTTTAAAGTAAAA	70.5%	0.0013420710	0.0015446480	ACICU_02910	putative surface adhesion protein	1359	1389
ATAATGGCTTTAATTGCTTACTTTAAGGGAA	70.5%	0.0045318430	0.0051792490	ACICU_00865	hypothetical protein	25	55
AATAATGGCTTTAATTGCTTACTTTAAGGGA	70.5%	0.0045318430	0.0051792490	ACICU_00865	hypothetical protein	24	54
TAATGGCTTTAATTGCTTACTTTAAGGGAAA	70.5%	0.0045318430	0.0051792490	ACICU_00865	hypothetical protein	26	56
CCTCTAAGAGATATCTTTTAATGAGCTTAGC	70.5%	0.0052323550	0.0054911600	ABTJ_02897	hypothetical protein	35	65
CTCTAAGAGATATCTTTTAATGAGCTTAGCC	70.5%	0.0052323550	0.0054911600	ABTJ_02897	hypothetical protein	36	66
ATGAATAGGGCTAAGCTCATTAAAAGATATC	70.5%	0.0052323550	0.0054911600	ABTJ_02897	hypothetical protein	44	74
AAGAGATATCTTTTAATGAGCTTAGCCCTAT	70.5%	0.0052323550	0.0054911600	ABTJ_02897	hypothetical protein	40	70
TAAGAGATATCTTTTAATGAGCTTAGCCCTA	70.5%	0.0052323550	0.0054911600	ABTJ_02897	hypothetical protein	39	69
AATAGGGCTAAGCTCATTAAAAGATATCTCT	70.5%	0.0052323550	0.0054911600	ABTJ_02897	hypothetical protein	41	71
ACATGAATAGGGCTAAGCTCATTAAAAGATA	70.5%	0.0052323550	0.0054911600	ABTJ_02897	hypothetical protein	46	76
AGGGCTAAGCTCATTAAAAGATATCTCTTAG	70.5%	0.0052323550	0.0054911600	ABTJ_02897	hypothetical protein	38	68
GAATAGGGCTAAGCTCATTAAAAGATATCTC	70.5%	0.0052323550	0.0054911600	ABTJ_02897	hypothetical protein	42	72
AGATATCTTTTAATGAGCTTAGCCCTATTCA	70.5%	0.0052323550	0.0054911600	ABTJ_02897	hypothetical protein	43	73
TCTAAGAGATATCTTTTAATGAGCTTAGCCC	70.5%	0.0052323550	0.0054911600	ABTJ_02897	hypothetical protein	37	67
ATATCTTTTAATGAGCTTAGCCCTATTCATG	70.5%	0.0052323550	0.0054911600	ABTJ_02897	hypothetical protein	45	75
AAGTTTATCACCACCGACTATTTGCAACAGT	70.5%	0.0104872900	0.0109121480	ABTJ_02588	hypothetical protein	162	192
TTTATCACCACCGACTATTTGCAACAGTGCC	70.5%	0.0104872900	0.0109121480	ABTJ_02588	hypothetical protein	159	189
GCACTGTTGCAAATAGTCGGTGGTGATAAAC	70.5%	0.0104872900	0.0109121480	ABTJ_02588	hypothetical protein	160	190
AGTTTATCACCACCGACTATTTGCAACAGTG	70.5%	0.0104872900	0.0109121480	ABTJ_02588	hypothetical protein	161	191

Table S2. List of strains

strain name	PATRIC ID	phenotype	ST	*bla* OXA-51	*bla* OXA-23	IS*Aba1*	Figure	Note	order in Figure S2
1462234	1310646.3	R	2	1	0	0	Figure 1 & S2		1
135867	1310629.3	R	2	1	1	0	Figure S2		2
1284800	1310627.3	R	2	1	1	0	Figure S2		3
470922	1310808.3	R	2	1	1	0	Figure S2		4
1043903	1310569.3	R	2	1	1	0	Figure S2		5
987421	1310559.3	R	2	1	1	0	Figure S2		6
23037	1310584.3	R	2	1	1	0	Figure S2		7
1291820	1310654.3	S	2	1	0	0	Figure 1 & S2		8
1146103	1310656.3	R	2	1	0	0	Figure 1 & S2		9
1440750	1310663.3	R	2	1	0	0	Figure 1 & S2		10
6935	1310575.3	R	2	1	0	0	Figure 1 & S2		11
1526966	1310675.3	R	2	1	0	0	Figure 1 & S2		12
1036938	1310554.3	R	2	1	0	0	Figure 1 & S2		13
58452	1310579.3	R	2	1	0	0	Figure 1 & S2		14
1247182	1310597.3	R	2	1	0	0	Figure 1 & S2		15
532413	1310577.3	R	2	1	0	0	Figure 1 & S2		16
1046051	1310570.3	R	2	1	0	0	Figure 1 & S2		17
1552389	1310812.3	S	2	1	0	1	Figure S2		18
1277411	1310655.3	S	2	1	0	0	Figure 1 & S2		19
1488685	1310807.3	S	2	1	0	0	Figure 1 & S2		20
323408	1310820.3	S	2	1	0	0	Figure 1 & S2		21
273929	1310738.3	R	2	1	0	0	Figure 1 & S2		22
1207552	1310730.3	R	2	1	0	0	Figure 1 & S2		23
23671	1310729.3	R	2	1	0	0	Figure 1 & S2		24
1539026	1310809.3	R	2	1	1	0	Figure S2		25
339786	1310801.3	S	2	1	0	1	Figure S2		26
145660	1310789.3	S	2	1	0	0	Figure 1 & S2		27
1428368	1310797.3	S	2	1	0	0	Figure 1 & S2		28
825610	1310722.3	S	2	1	1	0	Figure S2		29
70136	1310787.3	S	2	1	0	0	Figure 1 & S2		30
1552865	1310679.3	S	2	1	0	0	Figure 1 & S2		31
1051176	1310583.3	S	2	1	0	0	Figure 1 & S2		32
11126	1310802.3	S	2	1	0	1	Figure S2		33
18689	1310573.3	R	2	1	0	0	Figure 1 & S2		34
564012	1310560.3	R	2	1	0	0	Figure 1 & S2		35
916567	1310568.3	R	2	1	0	0	Figure 1 & S2		36
15827	1310631.3	R	2	1	1	0	Figure S2		37
947299	1310587.3	R	2	1	1	0	Figure S2		38
34654	1310581.3	S	670	1	0	0	Figure 1 & S2		39
400834	1310616.3	R	2	1	1	0	Figure S2		40
7893	1310810.3	R	2	1	0	0	Figure 1 & S2		41
1271651	1310732.3	R	2	1	1	0	Figure S2		42
1178044	1310731.3	R	2	1	1	0	Figure S2		43
98826	1310793.3	S	2	1	0	0	Figure 1 & S2		44
628418	1310755.3	R	2	1	0	0	Figure 1 & S2		45
1266220	1310758.3	R	2	1	0	0	Figure 1 & S2		46
1465485	1310649.3	R	2	1	1	0	Figure S2		47
213697	1310763.3	R	2	1	1	0	Figure S2		48
2887	1310754.3	R	2	1	0	0	Figure 1 & S2		49
647609	1310610.3	R	2	1	1	0	Figure S2		50
1095464	1310747.3	R	2	1	1	0	Figure S2		51
981176	1310792.3	R	2	1	0	0	Figure 1 & S2		52
1117819	1310780.3	R	2	1	0	0	Figure 1 & S2		53
73736	1310741.3	R	2	1	1	0	Figure S2		54
959073	1310557.3	R	2	1	1	0	Figure S2		55
1064293_46	1310825.3	R	2	1	0	0	Figure 1 & S2		56
1482820	1310716.3	R	671	1	0	0	Figure 1 & S2		57
942133	1310561.3	R	2	1	0	0	Figure 1 & S2		58
1045092	1310739.3	R	2	1	1	0	Figure S2		59
1159076	1310644.3	R	2	1	1	0	Figure S2		60
554515	1310672.3	S	2	1	0	0	Figure 1 & S2		61
1031433	1310586.3	R	2	1	1	0	Figure S2		62
1232509	1310774.3	R	2	1	0	0	Figure 1 & S2		63
1426993	1310805.3	R	2	1	1	0	Figure S2		64
993520	1310555.3	R	2	1	1	0	Figure S2		65
136706	1310558.3	R	2	1	1	0	Figure S2		66
1294217	1310620.3	R	2	1	1	0	Figure S2		67
1406750	1310617.3	R	2	1	1	0	Figure S2		68
1397084	1310614.3	R	NA	1	1	0	Figure S2		69
1406182	1310796.3	R	2	1	1	0	Figure S2		70
1593273	1310698.3	R	2	1	1	0	Figure S2		71
1046674	1310567.3	R	2	1	0	0	Figure 1 & S2		72
17534	1310556.3	R	2	1	0	0	Figure 1 & S2		73
1287985	1310746.3	R	195	1	1	0	Figure S2		74
1552818	1310666.3	R	2	1	1	0	Figure S2		75
755829	1310757.3	R	2	1	0	1	Figure S2		76
1457504	1310717.3	R	2	1	1	0	Figure S2		77
1051830	1310585.3	R	523	1	1	0	Figure S2		78
756476	1310815.3	R	664	1	0	0	Figure 1 & S2		79
923118	1310708.3	R	2	1	1	0	Figure S2		80
724909	1310650.3	R	2	1	1	0	Figure S2		81
458282	1310811.3	R	2	1	1	0	Figure S2		82
1392509	1310775.3	R	2	1	0	0	Figure 1 & S2		83
1494580	1310799.3	R	2	1	1	0	Figure S2		84
1264936	1310743.3	R	2	1	1	0	Figure S2		85
836190	1310727.3	R	2	1	1	0	Figure S2		86

655555	1310712.3	R	2	1	1	0	Figure S2		87
1022959	1310751.3	R	2	1	0	1	Figure S2		88
1296252	1310753.3	R	2	1	1	0	Figure S2		89
1294222	1310765.3	R	2	1	0	0	Figure 1 & S2		90
3390	1310710.3	S	10	1	0	0	Figure 1 & S2		91
466760	1310611.3	S	10	1	0	0	Figure 1 & S2		92
1007214	1310784.3	S	10	1	0	0	Figure 1 & S2		93
50595	1310662.3	S	10	1	0	0	Figure 1 & S2		94
1542444	1310681.3	S	675	1	0	0	Figure S2	different species	95
1295259	1310608.3	S	119	1	0	0	Figure S2	different species	96
742879	1310791.3	S	NA	1	0	0	Figure S2	different species	97
826659	1310764.3	S	NA	1	0	0	Figure S2	different species	98
1245249	1310724.3	S	NA	1	0	0	Figure S2	different species	99
1564232	1310723.3	S	NA	1	0	0	Figure S2	different species	100
883425	1310773.3	S	220	1	0	0	Figure S2	different species	101
907131	1310670.3	S	220	1	0	0	Figure S2	different species	102
809848	1310637.3	S	666	1	0	0	Figure S2	different species	103
723929	1310711.3	S	93	1	0	0	Figure S2	different species	104
1289694	1310609.3	S	93	1	0	0	Figure S2	different species	105
1451147	1310642.3	S	NA	1	0	0	Figure S2	different species	106
225588	1310788.3	S	NA	1	0	0	Figure S2	different species	107
72431	1310685.3	S	93	1	0	0	Figure S2	different species	108
1475718	1310652.3	S	93	1	0	0	Figure S2	different species	109
478810	1310720.3	R	93	1	0	0	Figure S2	different species	110
1264765	1310824.3	S	214	1	0	0	Figure S2	different species	111
1294243	1310756.3	S	214	1	0	0	Figure S2	different species	112
1578804	1310689.3	S	667	1	0	0	Figure S2	different species	113
573719	1310759.3	S	563	1	0	0	Figure S2	different species	114
1288284	1310709.3	R	NA	1	1	0	Figure S2	different species	115
1437282	1310638.3	S	NA	0	0	0	Figure S2	different species	116
146457	1310623.3	S	NA	0	0	0	Figure S2	different species	117
479375	1310601.3	S	NA	0	0	0	Figure S2	different species	118
983759	1310660.3	S	NA	0	1	0	Figure S2	different species	119
230853	1310651.3	S	NA	0	1	0	Figure S2	different species	120
1239920	1310680.3	S	NA	0	1	0	Figure S2	different species	121
272263	1310639.3	S	NA	0	1	0	Figure S2	different species	122
1294596	1310603.3	S	NA	0	1	0	Figure S2	different species	123
1461402	1310647.3	S	NA	0	1	0	Figure S2	different species	124
869535	1310621.3	S	NA	0	1	0	Figure S2	different species	125
348935	1310605.3	S	NA	0	0	0	Figure S2	different species	126
1130196	1310772.3	S	395	0	0	0	Figure S2	different species	127
1566109	1310683.3	S	395	0	0	0	Figure S2	different species	128
694762	1310705.3	S	410	0	0	0	Figure S2	different species	129
1396970	1310615.3	S	410	0	0	0	Figure S2	different species	130
1281984	1310795.3	S	68	0	0	0	Figure S2	different species	131
1245593	1310728.3	S	68	0	0	0	Figure S2	different species	132
259052	1310695.3	S	71	0	0	0	Figure S2	different species	133
6112	1310669.3	S	71	0	0	0	Figure S2	different species	134
88816	1310761.3	S	71	0	0	0	Figure S2	different species	135
216872	1310734.3	S	530	0	0	0	Figure S2	different species	136
1179249	1310790.3	S	768	0	0	0	Figure S2	different species	137
766875	1310677.3	S	224	0	0	0	Figure S2	different species	138
1424608	1310779.3	S	279	0	0	0	Figure S2	different species	139
21871	1310626.3	S	279	0	0	0	Figure S2	different species	140
1571545	1310816.3	S	NA	0	0	0	Figure S2	different species	141
144107	1310688.3	S	154	1	0	0	Figure 1 & S2		142
1040094	1310590.3	S	416	1	1	0	Figure S2		143
1075025	1310596.3	R	514	1	1	0	Figure S2		144
1039621	1310592.3	R	416	1	1	0	Figure S2		145
42887	1310691.3	R	514	1	1	0	Figure S2		146
43926	1310676.3	R	514	1	1	0	Figure S2		147
942194	1310591.3	R	650	1	0	0	Figure 1 & S2		148
562700	1310588.3	R	416	1	1	0	Figure S2		149
1202252	1310624.3	R	416	1	0	0	Figure 1 & S2		150
1058283	1310771.3	R	416	1	0	0	Figure 1 & S2		151
1032241	1310571.3	R	527	1	0	0	Figure 1 & S2		152
1188188	1310742.3	R	500	1	0	0	Figure 1 & S2		153
480175	1310745.3	R	500	1	0	0	Figure 1 & S2		154
1237893	1310744.3	R	500	1	0	0	Figure 1 & S2		155
29280	1310595.3	R	522	1	1	0	Figure S2		156
1271213	1310737.3	R	500	1	0	0	Figure 1 & S2		157
940793	1310633.3	R	500	1	0	0	Figure 1 & S2		158
1406589	1310760.3	R	521	1	0	0	Figure 1 & S2		159
299505	1310643.3	S	508	1	0	0	Figure 1 & S2		160
21072	1310697.3	S	336	1	0	0	Figure 1 & S2		161
1440422	1310641.3	S	203	1	0	0	Figure 1 & S2		162
4749	1310634.3	S	503	1	0	0	Figure 1 & S2		163
232184	1310694.3	S	16	1	0	0	Figure 1 & S2		164
1043794	1310564.3	R	16	1	0	0	Figure 1 & S2		165
972082	1310563.3	S	16	1	0	0	Figure 1 & S2		166
1064293_45	1310750.3	S	16	1	0	0	Figure 1 & S2		167
268680	1310701.3	S	16	1	0	0	Figure 1 & S2		168
1297	1310580.3	R	537	1	1	1	Figure S2		169
83444	1310684.3	S	533	1	0	0	Figure 1 & S2		170
1106579	1310659.3	S	505	1	0	0	Figure 1 & S2		171
ATCC 17978	ATCC17978	S	437	1	0	0	Figure 1 & S2	complete genome	172
1598530	1310700.3	S	56	1	0	0	Figure 1 & S2		173
532279	1310692.3	S	519	1	0	0	Figure 1 & S2		174
99063	1310630.3	S	506	1	0	0	Figure 1 & S2		175
625974	1310607.3	S	509	1	0	0	Figure 1 & S2		176
1429530	1310636.3	S	25	1	0	0	Figure 1 & S2		177
984213	1310604.3	S	25	1	0	0	Figure 1 & S2		178
233846	1310736.3	S	126	1	0	0	Figure 1 & S2		179
1419130	1310619.3	S	529	1	0	0	Figure 1 & S2		180
121738	1310818.3	S	138	1	0	0	Figure 1 & S2		181
1295743	1310613.3	S	240	1	0	0	Figure 1 & S2		182

846928	1310748.3	S	535	1	0	0	Figure 1 & S2		183
1525283	1310665.3	S	32	1	0	0	Figure 1 & S2		184
781407	1310599.3	S	32	1	0	0	Figure 1 & S2		185
1391434	1310762.3	S	285	1	0	0	Figure 1 & S2		186
118362	1310618.3	S	241	1	0	0	Figure 1 & S2		187
1035119	1310622.6	S	536	1	0	0	Figure 1 & S2		188
541915	1310786.3	S	532	1	0	0	Figure 1 & S2		189
1062314	1310725.3	R	54	1	0	0	Figure 1 & S2		190
277047	1310781.3	S	652	1	0	0	Figure 1 & S2		191
1295549	1310770.3	S	652	1	0	0	Figure 1 & S2		192
426863	1310778.3	S	652	1	0	0	Figure 1 & S2		193
1297549	1310606.3	S	528	1	0	0	Figure 1 & S2		194
831240	1310702.3	S	78	1	0	0	Figure 1 & S2		195
855125	1310661.3	S	78	1	0	0	Figure 1 & S2		196
1096934	1310657.3	S	78	1	0	0	Figure 1 & S2		197
1267820	1310628.3	S	103	1	0	0	Figure 1 & S2		198
14216	1310798.3	R	499	1	0	0	Figure 1 & S2		199
1533268	1310721.3	R	499	1	0	0	Figure 1 & S2		200
1170863	1310782.3	R	499	1	0	0	Figure 1 & S2		201
397971	1310686.3	R	499	1	0	0	Figure 1 & S2		202
1413735	1310776.3	R	499	1	0	0	Figure 1 & S2		203
457946	1310740.3	R	499	1	0	0	Figure 1 & S2		204
607805	1310719.3	S	499	1	0	0	Figure 1 & S2		205
214216	1310783.3	R	499	1	0	0	Figure 1 & S2		206
1195185_80	1310826.3	R	499	1	0	0	Figure 1 & S2		207
722310	1310813.3	R	499	1	0	0	Figure 1 & S2		208
1499986	1310673.3	R	499	1	0	0	Figure 1 & S2		209
951631	1310653.3	R	499	1	0	0	Figure 1 & S2		210
754286	1310668.3	R	499	1	0	0	Figure 1 & S2		211
496487	1310625.3	R	499	1	0	0	Figure 1 & S2		212
1293320	1310752.3	S	49	1	0	0	Figure 1 & S2		213
1461963	1310645.3	S	49	1	0	0	Figure 1 & S2		214
1546444	1310674.3	S	NA	1	0	0	Figure 1 & S2		215
1417041	1310703.3	S	108	1	0	0	Figure 1 & S2		216
342950	1310640.3	S	512	1	0	0	Figure 1 & S2		217
318814	1310707.3	S	651	1	0	0	Figure 1 & S2		218
1412924	1310706.3	S	510	1	0	0	Figure 1 & S2		219
1475764	1310718.3	R	2	1	0	0	Figure 1 & S2		220
1505311	1310806.3	R	NA	1	1	0	Figure S2		221
737393	1310735.3	R	2	1	1	0	Figure S2		222
1032359	1310572.3	R	2	1	0	0	Figure 1 & S2		223
96512	1310602.3	R	2	1	1	0	Figure S2		224
10519	1310749.3	R	2	1	0	0	Figure 1 & S2		225
303002	1310794.3	R	2	1	1	0	Figure S2		226
1471012	1310713.3	R	2	1	1	0	Figure S2		227
1289546	1310600.3	R	2	1	0	0	Figure 1 & S2		228
1237202	1310767.3	R	2	1	1	0	Figure S2		229
466215	1310785.3	R	2	1	1	0	Figure S2		230
1575710	1310699.3	R	2	1	1	0	Figure S2		231
1397513	1310768.3	R	2	1	0	0	Figure 1 & S2		232
34001	1310578.3	R	2	1	1	0	Figure S2		233
UH16108	1398959.3	R	2	1	0	0	Figure 1		
UH19608	1398962.3	R	79	1	0	0	Figure 1		
UH3807	1398970.3	R	2	1	0	0	Figure 1		
UH6907	1398978.3	R	79	1	0	0	Figure 1		
ACICU	405416.6	R	2	1	0	0	Figure 1	complete genome	
AC29	470.1288	R	2	1	0	0	Figure 1	complete genome	
MRSN16881	470.1553	R	10	1	0	0	Figure 1		
MRSN16882	470.1554	R	10	1	0	0	Figure 1		
UH588_663	470.627	R	2	1	0	0	Figure 1		
UH367_558	470.635	R	NA	1	0	0	Figure 1		
UH106_447	470.659	R	NA	1	0	0	Figure 1		
UH384_444	470.661	R	NA	1	0	0	Figure 1		
UH535_423	470.672	R	NA	1	0	0	Figure 1		
UH535_419	470.673	R	2	1	0	0	Figure 1		
UH535_418	470.674	R	2	1	0	0	Figure 1		
UH293_373	470.685	R	2	1	0	0	Figure 1		
UH126_102	470.733	R	2	1	0	0	Figure 1		
UH455_38	470.762	R	2	1	0	0	Figure 1		
UH348_25	470.765	R	2	1	0	0	Figure 1		
UH455_6	470.768	R	2	1	0	0	Figure 1		
AB030	-	R	79	1	0	0	Figure 1	complete genome	
MDR-TJ	889738.3	R	2	1	0	0	Figure 1	complete genome	
